# A new generation of mesenchymal stromal/stem cells differentially trained by immunoregulatory probiotics in a lupus microenvironment

**DOI:** 10.1186/s13287-023-03578-z

**Published:** 2023-12-10

**Authors:** Akram Hoseinzadeh, Mahmoud Mahmoudi, Houshang Rafatpanah, Zahra Rezaieyazdi, Jalil Tavakol Afshari, Sara Hosseini, Seyed-Alireza Esmaeili

**Affiliations:** 1 Immunology Research Center, Mashhad University of Medical Sciences, Mashhad, Iran; 2 Immunology Department, Faculty of Medicine, Mashhad University of Medical Sciences, Mashhad, Iran; 3 Immunology Research Centre, Division of Inflammation and Inflammatory Diseases, Mashhad University of Medical Sciences, Mashhad, Iran; 4 Rheumatic Diseases Research Center, Mashhad University of Medical Sciences, Mashhad, Iran; 5 Faculty of Medicine, Department of Immunology, BuAli Research Institute, Mashhad University of Medical Sciences, Mashhad, Iran; 6 Student Research Committee, Mashhad University of Medical Sciences, Mashhad, Iran

**Keywords:** Mesenchymal stem/stromal cells, Probiotics, Lupus nephritis, Cytokines, T cells

## Abstract

**Background:**

Increasing evidence suggests that multipotent mesenchymal stem/stromal cells (MSCs) are a promising intervention strategy in treating autoimmune inflammatory diseases. It should be stated that systemic immunoregulation is increasingly recognized among the beneficial effects of MSCs and probiotics in treating morbid autoimmune disorders such as lupus. This study aimed to determine if immunoregulatory probiotics L. rhamnosus or L. delbrueckii can change the immunomodulatory effects of MSCs in lupus-like disease.

**Methods:**

Pristane-induced lupus (PIL) mice model was created via intraperitoneal injection of Pristane and then confirmed. Naïve MSCs (N-MSCs) were coincubated with two Lactobacillus strains, rhamnosus (R-MSCs) or delbrueckii (D-MSCs), and/or a combination of both (DR-MSCs) for 48 h, then administrated intravenously in separate groups. Negative (PBS-treated normal mice) and positive control groups (PBS-treated lupus mice) were also investigated. At the end of the study, flow cytometry and enzyme-linked immunosorbent assay (ELISA) analysis were used to determine the percentage of Th cell subpopulations in splenocytes and the level of their master cytokines in sera, respectively. Moreover, lupus nephritis was investigated and compared. Analysis of variance (ANOVA) was used for multiple comparisons.

**Results:**

Abnormalities in serum levels of anti-dsDNA antibodies, creatinine, and urine proteinuria were significantly suppressed by MSCs transplantation, whereas engrafted MSCs coincubation with both L. strains did a lesser effect on anti-dsDNA antibodies. L. rhamnosus significantly escalated the ability of MSCs to scale down the inflammatory cytokines (IFN-ɣ, IL-17), while L. delbrueckii significantly elevated the capacity of MSCs to scale down the percentage of Th cell subpopulations. However, incubation with both strains induced MSCs with augmented capacity in introducing inflammatory cytokines (IFN-ɣ, IL-17). Strikingly, R-MSCs directly restored the serum level of TGF-β more effectively and showed more significant improvement in disease parameters than N-MSCs. These results suggest that R-MSCs significantly attenuate lupus disease by further skew the immune phenotype of MSCs toward increased immunoregulation.

**Conclusions:**

Results demonstrated that Lactobacillus strains showed different capabilities in training/inducing new abilities in MSCs, in such a way that pretreated MSCs with L. rhamnosus might benefit the treatment of lupus-like symptoms, given their desirable properties.

**Graphical abstract:**

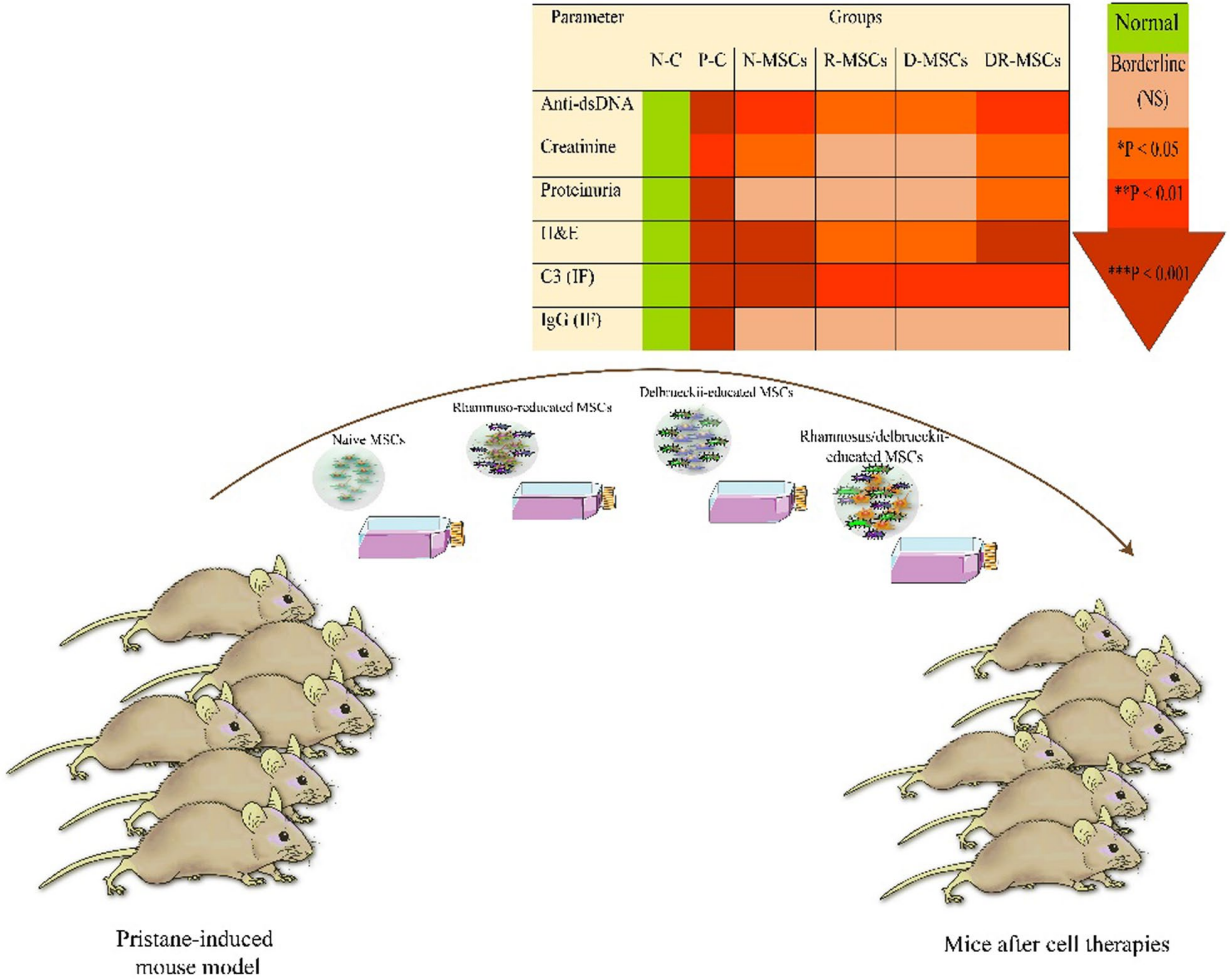

## Introduction

A subpopulation of somatic cells is bone marrow-derived mesenchymal progenitors as multipotent, self-renewing, and non-immunogenic mesenchymal stem/stromal cells (MSCs) [1, 2]. Accumulating data shows that MSCs are present in almost all tissues [3]. MSCs are a unique type of cells in a rest state (naïve MSCs) that track the “cues” from the inflamed and injured niches, migrate by adhering and spreading on the walls of blood vessels and tissues, sensing activation signals from pro-inflammatory cytokines on the move and following homing and incorporating into the integral components of the organ, interact with cell/cell products which leads to inducing specific activation signaling patterns and ultimately dictating MSC phenotype [3–5]. Naïve MSCs can be altered in their properties and converted into pro/anti-inflammatory population cells. The master regulator IFN-ɣ (at high concentration) is sufficient to instruct the precursors to commit to the anti-inflammatory fate; however, TNF-α and IL-1 have synergistic effects [1, 6]. Once activated, MSCs have shown capability in mutual communication with the components of innate and adaptive arms of the immune through direct (cell–cell contact or their soluble factors) and/or indirect mechanisms. Immunomodulatory MSCs provide their cognate diverse myeloid and lymphoid-lineage cells with the essential help needed to induce reprogramming through several cellular signaling pathways, followed by alteration in gene transcription and, ultimately, dedifferentiation of immune cells into distinct phenotype. The focus of increased research interest is the MSC2 exhibiting pronounced anti-inflammatory activity; however, MSCs1, which otherwise help exacerbate inflammation by the immune system, can be wrathful [7, 8]. Identifying biological effectors, physiologic conditions and specific signaling networks that govern desired differentiation programs or prevent spurious differentiation of MSCs, might inform novel strategies of selective therapeutic intervention for practical clinical application [9]. In recent years, immunologists have shown increasing interest in using MSCs for adoptive cell therapy and have predicted a bright future for MSC-directed therapy in regulating immune response-associated inflammation, cancer therapy, and tissue regeneration. Aberrant immune response patterns have been evident in physiological and pathological settings such as autoimmune diseases [10]. Recently, MSCs transplantation as a new strategy and trials of alternative therapies have been used to resolve toxicities of currently used drugs, such as immunosuppressive agents and corticosteroids. In an expanding body of studies, the communication mechanism between MSCs and bioactive molecules to increase the limited immunoregulatory capacity of MSCs has attracted increasing attention and has emerged as a promising platform f to enhance and extend the therapeutic application of MSCs [1, 11, 12]. Recently, Amendola et al. have addressed that proteins, carbohydrates, and lipids act differently on MSCs bearing on the modulation of gene expression and controlling the fate of cell lineages and differentiation of them [13]. Researchers are learning to use engineered-MSCs as well as MSCs as direct or indirect payload carriers; however, considering undesirable side effects and their limitations, using immunoregulatory probiotics and/or their metabolites could be a new perspective. In this regard, of the unique characteristics of MSCs [14], we were actively looking for ways to increase the immunoregulatory activity of them. Current studies showed that MSCs express functional pattern recognition receptors and interact dynamically with various bacterial structures and their associated molecules [15–19]. The interplay between bacteria and MSCs also determines whether administered MSCs adopt an anti-inflammatory or pro-inflammatory immunophenotype [1, 20–22]. Beneficial bacteria (probiotics) are defined by the Food and Agriculture Organization of the United Nations (FAO) and World Health Organization (WHO) as non-pathogenic living microorganisms that can benefit the host when provided in sufficient quantities [23, 24]. It should be noted that probiotics and the host’s immune system have shown their interdependence in developing and restoring each other’s desired functions in various conditions [25, 26]. Probiotic therapy (the average recommended dose for each injection is 10^9^–10^11^ microorganisms [27]) is one of the several approaches that has gained interest worldwide in the treatment of inflammatory diseases, cancer, and wound healing [12, 25, 28–33]. The therapeutic rationale is based on the role of probiotics in regulating immune cell development, immune tolerance control and autoimmunity, maintenance of immune homeostasis, and required for normal development and maturation of specific lymphoid tissue [1, 34–40]. Numerous probiotic therapies, including Lactobacillus strains, have been reported in experimental inflammatory-mediated diseases such as lupus, allergies, and arthritis [30–33, 41–44]. The genus Lactobacillus spp. (over 160 species), a well-known probiotic, live in close contact with humans in the gastrointestinal tract, vagina, and oral cavity, but not all are beneficial as potential probiotics for immunotherapy [45, 46]. The Lactobacilli, utilized widely for the production of fermented foods, are believed to be one of the most abundant beneficial bacteria found in the microbiota of the human gut [47]. Different strains of Lactobacillus have been examined experimentally and mechanically to determine their potential efficacy in managing cancer, metabolic diseases, and autoimmune disorders. In a similar vein, systemic lupus erythematosus (SLE) has been the focus of both clinical and experimental trials [48]. Systemic lupus erythematosus (SLE) is an autoimmune disease characterized by the production of pathogenic autoantibodies to components of the cell nucleus. As a result of self-antigen encounters, including components of the cell nucleus such as double-stranded DNA (dsDNA) with the immune system, the host self-reactive B and T cells synthesize and secrete pathogenic autoantibodies and release cytokines, respectively, attracting inflammatory immune cells to the site of immunocomplexes formation that trigger chronic inflammatory damage in multiorgan systems [49–51]. Lactobacillus abundance varies depending on the SLE animal models, which might be important in the development of SLE. Of particular note is the deficiency of the probiotic genera Lactobacillus in the microbiota of SLE in some cases [47]. Emerging evidence suggests that Lactobacillus spp. can act as an environmental agent with beneficial therapeutic and anti-inflammatory effects on SLE, possess the ability to alleviate lupus-like disease, and provide evidence for further microbiota-targeted therapies [48, 52, 53]. For instances, recent research found that certain species of Lactobacilli, specifically L. delbrueckii and L. rhamnosus, possess the capability to modulate the expression levels of inflammatory/suppressive agents, which could be beneficial in the management of SLE patients [54–56]. Also, it has shown that Lactobacillus could cling to various immune/non-immune cells, interact with them and regulate their response to their microenvironment [55, 57]. Moreover, recently generated evidence by Zhang et al., and Cabana-Puig et al., have suggested that the Lactobacillus spp. including L. rhamnosus has reduced proteinuria and autoantibodies, attenuated kidney inflammation, splenomegaly, and lymphadenopathy in lupus-prone mice [53, 58].

MSCs and probiotics can influence multicellular gene networks in their microenvironment; however, how MSCs interact with probiotics still needs to be fully understood [1, 59, 60]. Transplanted MSCs have shown significant contributions in restoring gut microbiome alteration and enhancing pathogenic bacterial eradication culminating, which results in enhanced functions of both microbiota and MSCs [25]. Some preclinical relevant studies with new perspectives of combined therapies are highlighted [61, 62]. On the other hand, another study has reported that probiotics can alter cytokine gene transcription and surface protein expressions, differentiation potential, migration, and inflammatory signaling pathways, by and large, improving the immunomodulation ability in MSCs [1, 25, 62, 63]. In addition, many authors have reported that the cross point between MSCs and probiotics is auspicious in experimental studies [63–65]. However, in vivo is a dynamic microenvironment whose components have regulatory effects regarding directing cellular signaling and differentiation. Based on the screening of L. rhamnosus and L. delbrueckii identification of probiotic characteristics [66], relief delivered by probiotic and/or MSCs to lupus mice models and its effect on lupus disease indicators provide a new theoretical basis for researching the treatment outcome of probiotic-educated MSCs. Therefore, the probiotic-educated MSCs were explored from the perspectives of animal experiments, pathology, and immunology.

## Materials and methods

### Isolation and identification of bone marrow mesenchymal stromal/stem cells

Mouse primary MSCs were initially isolated from bone aspirates of 6- to 8-wk-old BALB/c, cultured, and identified precisely as previously reported [5]. In brief, femurs and tibias were isolated, cleaned from any remaining flesh, and flushed with culture media (low glucose Dulbecco’s modified Eagle’s medium (DMEM) with 1% penicillin/streptomycin and 15% fetal bovine serum, two mM L-glutamine) to extract the bone aspirates. It was plated for three days in 25 cm^2^ cell culture bottles with 5% CO2 and 37 °C. Fresh culture medium was added every 3–4 days until the culture reached 80% confluence. Cells were passaged once they reached 80% confluency. All experiments were conducted using MSCs in passages 3–5. MSCs are characterized by adherence to plastic, expression of the surface molecules CD73, CD90, and CD105 without CD34, CD45, and CD11b surface molecules, and ability to differentiate into adipocytes and osteocytes mice, as previously shown [5].

### Bacteria and growth conditions

Bacterial strains in the current experimental study, L. rhamnosus ATCC9595, and L. delbrueckii PTCC1743 subsp. Lactis were obtained from the Pasteur Institute of Iran and the Iranian Research Organization for Science and Technology. The probiotic strains were cultured per a previously published protocol [67]. Briefly, the Lactobacillus strain was cultured in De Man, Rogosa, and Sharpe (MRS) broth under anaerobic conditions at 37 °C. Before use, all bacterial cultures were centrifuged, washed twice with phosphate-buffered saline (PBS) solution, and resuspended in DMEM without penicillin/streptomycin, to the desired CFU/mL doses for in vitro experiments. After washing twice in a PBS solution, the number of probiotics was calculated, and 10^9^ bacteria/10^6^ MSCs were prepared and then coincubated for 48 h.

## MSC/bacteria association

To investigate whether the incubation of probiotics could alter MSCs treatment outcomes, MSCs were cultured with probiotics at MSC: probiotic ratio of 10^6^:10^9^ for two days. In the third passage of MSCs, when the cells reached 80%–90% confluency, the culture supernatant was discarded, cells were washed twice with PBS and replaced by an addition of DMEM low glucose media without P/S and incubated at 37˚C in 5% CO2 humidified incubator. Bacteria were collected at the second-transfer mid-log phase. Bacteria were centrifuged, growth media were aspirated off, bacterial strains were washed twice with PBS, and pellets were suspended in DMEM low glucose media without penicillin/streptomycin. L. rhamnosus or L. delbrueckii were adjusted to a concentration of 10^9^ CFU/mL before adding each microbe suspension independently [multiplicity of infection (MOI) 1:1000] to the MSCs [1]. MSCs were coincubated with L. rhamnosus (as R-MSCs) or L. delbrueckii (as D-MSCs) and/or a mix (as DR-MSCs) of them (equal numbers of L. rhamnosus and L. delbrueckii at the same ratio and at the same time) for 48 h at 37 °C and 5% CO2. After incubation, MSCs were alive when viewed with light microscopy and did not show any morphologic changes (all cells were homogeneous and exhibited a spindle-shaped morphology); all probiotics were washed away, MSCs monolayer was washed twice with PBS, harvested by trypsinization with Trypsin/EDTA (Invitrogen, USA), and then prepared for injection at a concentration of 10^9^ cells/150 µl PBS/mice.

### Mice

Conventional female BALB/c mice (Pasteur Institute, Tehran, Iran), six weeks of age, inbred, matched age (16 to 17 gr), were raised and maintained in pathogen-free and environmentally controlled room (22 ± 2 °C, 55 ± 5% RH) under a 12 h light/12 h dark cycle in the conventional experimental animal facility of the BuAli Research Institute, Mashhad, Iran. Mice chow and water were provided and administered on stock diet ad libitum. Before commencing the experiment, all animals were acclimatized for two weeks to adapt to their new environment, ascertain their physical well-being, and exclude any diseased or infected animals. At the end of the experiments, mice were killed by cervical dislocation. The Mashhad University of Medical Sciences Animal Care Committee approved this study.

### PIL mouse model induction

To establish the therapeutic scheme for our study, a single dose, 0.5 ml, intraperitoneally of Pristane, (Sigma Chemical Co.) (Molecular Weight 268.5 g/mol, computed by PubChem 2.1 (PubChem release 2021.05.07)), was administered to naïve mice on day 0 as previously described since Pristane is known to induce SLE-like disease [5].

### Experimental groups and treatment protocol

**Fig. 1:**
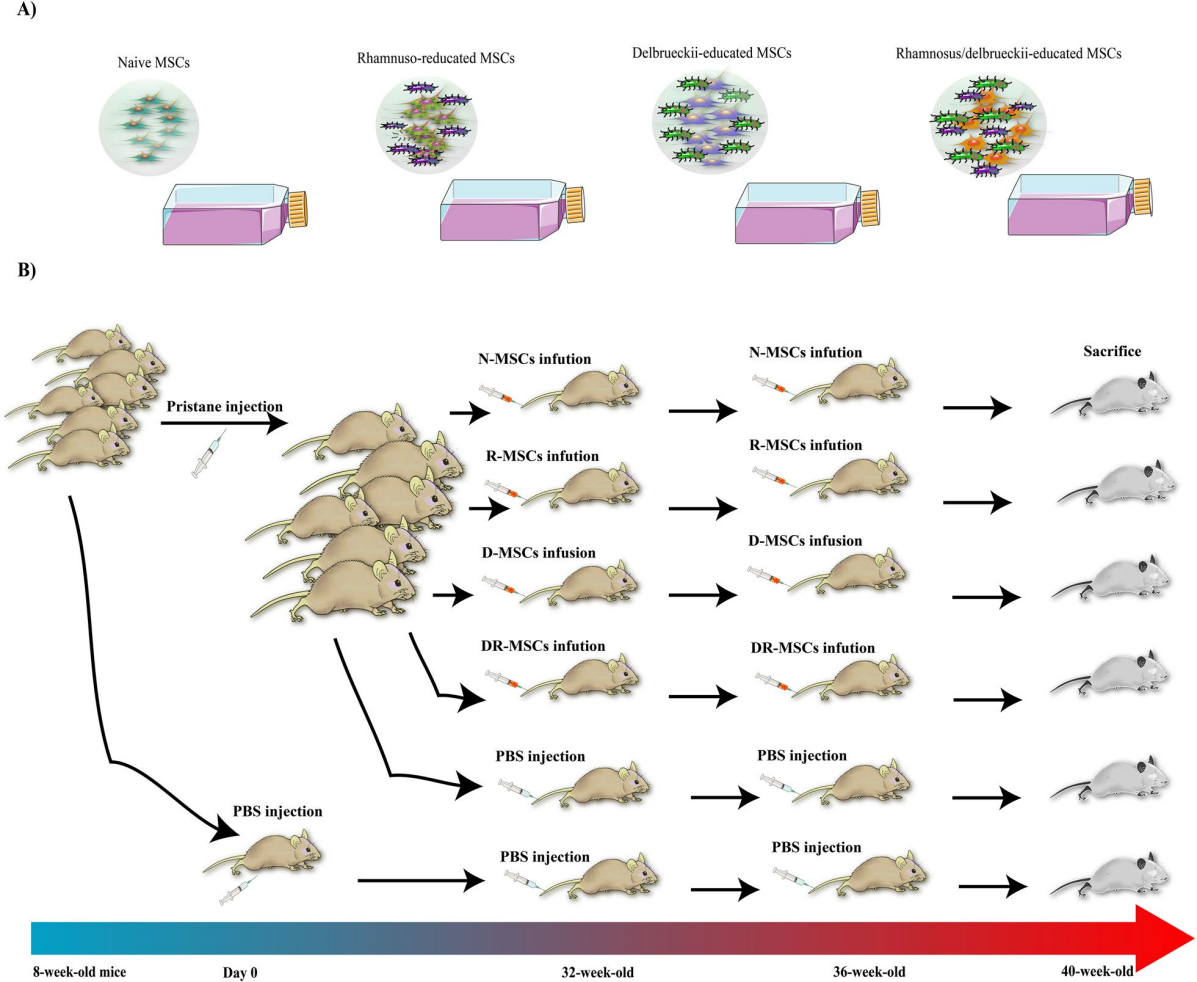
The scheme of lupus induction and allogenic BM-MSC transplantation procedure. 8-week-old BALB/c mice were used to induce lupus. **A**. Naïve MSCs were pretreated according to the protocol in the material method section. **B**. According to figure, MSCs (1 × 10^6^ cells/mouse) were transplanted in two dosages after lupus induction through the tail vein (at 32 and 36 weeks old, respectively), and mice were killed at 40 weeks of age

Six months post-Pristane injection, mice were randomly assigned into five groups (with six animals each) to administer naïve MSCs, R-MSCs, D-MSCs, DR-MSCs, or PBS. Age-matched naïve mice were used as normal controls. Cell transplantation was performed by monthly intravenous injections of cell suspensions containing 10^6^ MSCs. Under general anesthesia, PIL mice received two doses of cell administration (at 32 weeks and 36 weeks) by tail vein puncture. At the same schedule, an equal volume of PBS (150 µL) was used in the positive and negative control groups (PIL mice and normal mice, respectively). All mice were euthanized by cervical dislocation, under anesthesia, with ketamine (200 mg/kg) and xylazine (20 mg/kg) at 40 weeks of age for further analysis (Fig. 1).

### Macroscopic analysis

Body weight was assessed at the start of the experiment, and the end of the study, and then weight change was compared between groups. As described previously, liver, kidney, and spleen samples were harvested and weighed at the end of the study.

### Laboratory evaluation of serum creatinine, anti-dsDNA antibodies, and proteinuria

As previously described [5], peripheral blood serum and urine samples were collected from all mice. To evaluate the effects of cell therapy on hallmark biomarkers of lupus disease and functional recovery of the kidney, the creatinine concentration in sera, anti-dsDNA antibodies, and urine protein levels was measured and analyzed before the beginning of the cell therapy protocol (6 months post-pristane injection) and at the end of the study.

### Microscopic (light and immunofluorescence) study of kidney tissue

As previously reported [5], groups of mice were euthanized at 40 weeks of age under anesthesia and intraperitoneal injection of a ketamine-xylazine cocktail; the kidneys were removed and fixed in 10% neutral-buffered formalin. The specimens’ Sects. (3–5 μm) were analyzed by light microscopy after paraffin embedding, followed by standard hematoxylin and eosin (H&E) staining. Inflammatory cells were estimated based on a semiquantitative scoring system. The following features were graded as previously described based on the presence and degree of cellular infiltration (0, normal; 1, 2, and 3, mild, moderate, and severe infiltration, respectively. Two blinded experts performed the analyses. As described previously [5], frozen kidney sections of 3–5 µm were stained with the following antibodies: FITC-conjugated goat anti-mouse IgG antibody or FITC-conjugated goat anti-mouse C3. The intensity of fluorescence was determined by observation fluorescence microscope. Subsequently, immunofluorescence analysis was quantified based on the fluorescence intensity’s presence and extent (0, none; 1, 2, and 3, mild, moderate, and severe).

### ELISA test, cytokines measurements in serum

As described previously [5], mice were anesthetized, allowing peripheral blood to be collected by heart puncture. The concentrations of specific cytokines (IFN-ɣ, IL-4, IL-17, and TGF-β) were determined by enzyme-linked immunosorbent assay (ELISA) kits.

### Flow cytometry analysis of splenic Th cell subsets

As previously described [5], splenocytes were isolated and counted with a hematocytometer. Treg cell percentage was evaluated using the manufacturer’s instruction mouse regulatory T cell staining kit. Briefly, aliquots of 10^6^ cells were used for each test tube. For surface antigen detection, the cells were labeled with 5 µL fluorochrome-conjugated monoclonal antibodies: (FITC-conjugated anti-mouse CD4 and PE-conjugated anti-mouse CD25). Cells were fixed and permeabilized for intracellular staining with a Fixation/Permeabilization working solution and subsequently incubated with PE-conjugated anti-mouse Foxp3. The percentages of Th1, Th2, and Th17 cells were estimated as described previously [5]: the cells were stained for intracellular cytokines with fluorescein isothiocyanate FITC-anti-CD4, PE-anti-IFN-γ (Th1), PE-anti-IL-4 (Th2), and APC-anti-IL-17 (Th17). Data were acquired using a FACSCalibur flow cytometer, and subsequently analyzed by FlowJo software.

### Data analysis

Analysis of variance [ANOVA] followed by post hoc Tukey’s test was used for multiple comparisons. In contrast, the Bonferroni test was applied to compare proteinuria, serum levels of creatinine, and anti-dsDNA antibodies. Results were expressed as the means ± standard deviation (SEM) and considered statistically verified when *P* < 0.05. The software package used for the analyses was the STATISTICAL PACKAGE FOR SOCIAL SCIENCES (SPSS). The Shapiro–Wilk test was performed to determine the normality of the data.

## Results

### BM-derived MSCs identified as MSCs

**Fig. 2:**
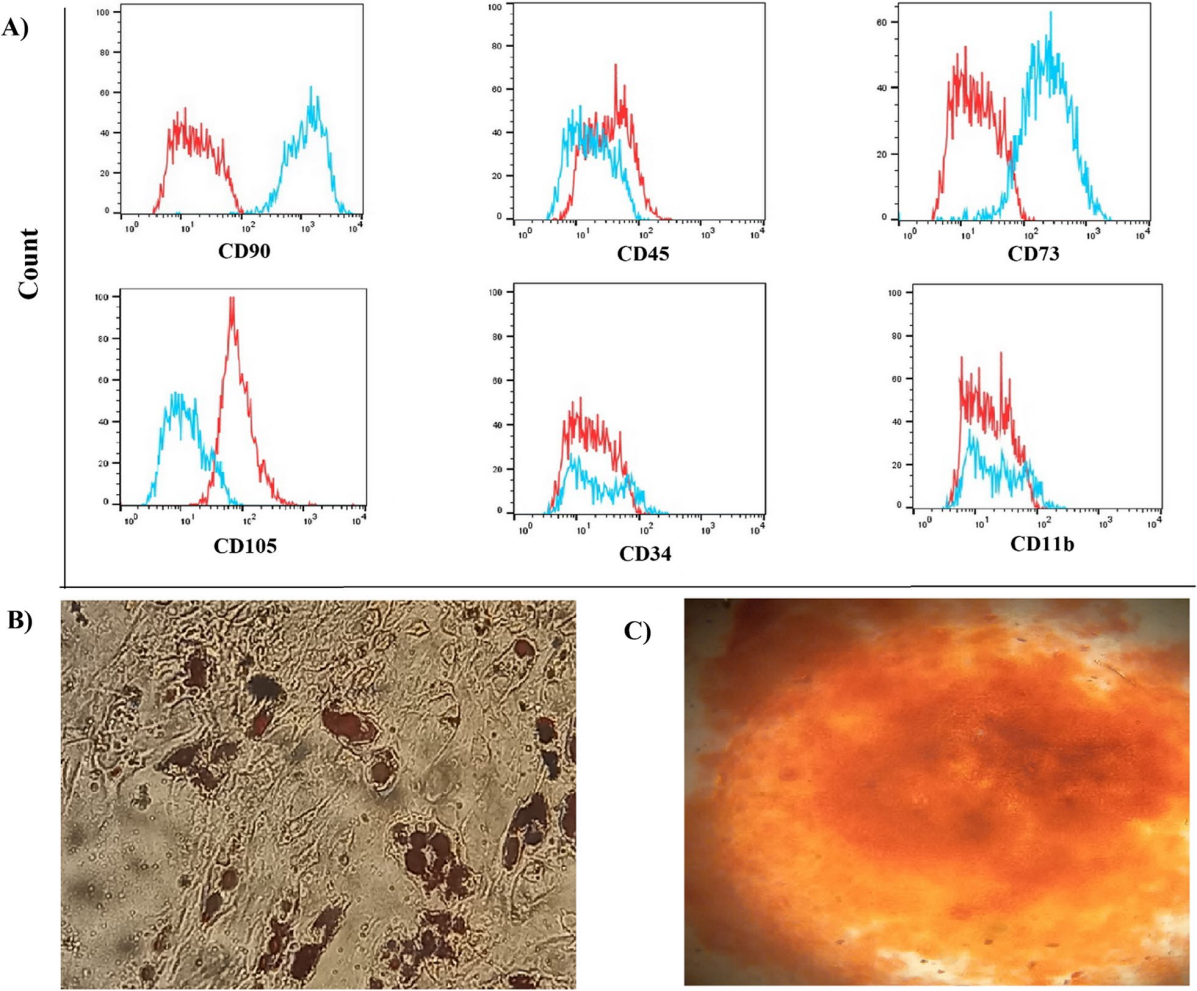
Bone marrow characterization of mice MSCs in vitro. Phenotype of BM-MSCs were assessed via flow cytometry analysis, which revealed that the cells expressed CD73, CD90, and CD105 and did not express the hematopoietic markers CD34, CD45, and CD11b **A**. Following 21 days of incubation in adipogenic medium, adipocytes were identified by accumulation of large fat droplets and staining for lipid with Oil Red O under light microscopy in cells. **A**. Also, the osteoblast differentiation of mice BM-MSCs was monitored under a light microscope **C**. At day 21, the calcified nodules of mice BM-MSCs were visualized by alizarin red S staining

BM-MSCs were isolated from healthy female BALB/c mice and characterized as previously shown [5], suggesting that nearly all of them fit the criterion for canonical MSCs. Colony-forming unit fibroblast (CFU-F) assay was performed as described previously. In brief, small, spindle-shaped or fibroblast-like rapidly self-renewing MSC populations, scoring negative for CD34, CD11b, and CD45 cells and positive for CD73, CD90, and CD105 (Fig. 2A), displaying commit to adipogenic, and osteogenic lineage fates when exposed to specific differentiation-inducing agents, were selected for further characterizations. Moreover, BM-MSCs were shown to have low adipogenic differentiation potential (Fig. 2B), whereas osteogenic differentiation was strong (Fig. 2C).

### Probiotic-trained MSCs treatment significantly reduced lymphadenopathy, but naïve MSCs treatment did not

SLE-like autoimmune disorder usually appears six months post-Pristane injection in BALB/c mice. To explore the effects of probiotic-pretreated MSCs interventions, we infused MSCs into PIL mice, either at the naïve state of the MSCs or at the pretreated with probiotics (Fig. 1). In contrast to normal mice, PIL mice developed nonmalignant lymphadenopathy associated with a characteristic expansion of autoreactive lymphocytes. Thus, we compared lipogranuloma lesions, as a secondary lymphoid organ, in order to investigate whether naïve MSCs or probiotic-pretreated MSCs treatment reduces lymphadenopathy. The expansion of these inflammatory lesions was significantly lower in probiotic-pretreated MSCs treated mice than in the P–C group, however, did not significant difference in those was found in the N-MSCs group compared with the P–C group (data not shown).

### Probiotic-trained MSCs changed the weight of the body, spleen, and liver

We quantified the weight of various organs, including the spleen, liver, and kidney. As shown in Table 1, the exposure of BALB/c mice to 0.5 ml Pristane led to significant increases in the weight of the spleen (*P* = 0.0021) and liver (*P* = 0.0018) than those in the N–C group. The administration of N-MSCs, R-MSCs, D-MSCs, and DR-MSCs diminished the weight of the spleen significantly from that measured in the P–C group, while the weight of the liver was not significantly affected. (Data are presented in Table 1.) Meanwhile, as shown in Table 1, the mean weight of the kidney was not significantly different among all six groups. Furthermore, no significant difference was observed in the mean body weight change at the end of the experiment between groups, despite clear signs of inflammation (lipogranuloma lesions) in the P–C group. (Data are presented in Table 1.) Collectively, the reduced cells in the spleen could impede inflammation and limit tissue damage.

**Table 1:** Effects of treatments on kidney, spleen, liver, and body weight of mice at the end of the study

Groups	Kidney (gr)	Spleen (gr)	Liver (gr)	Bodyweight (gr)
N–C	0.156 ± 0.0073	0.1340 ± 0.005	1.164 ± 0.005	27.96 ± 0.1958
P–C	0.158 ± 0.0047	0.5160 ± 0.0269	1.594 ± 0.035	29.72 ± 0.4164
N-MSCs	0.155 ± 0.0041	0.2520 ± 0.012	1.660 ± 0.0594	29.34 ± 0.3586
R-MSCs	0.156 ± 0.0086	0.1680 ± 0.0139	1.464 ± 0.0320	28.96 ± 0.2839
D-MSCs	0.157 ± 0.0015	0.1600 ± 0.0070	1.522 ± 0.0354	28.92 ± 0.3813
DR-MSCs	0.156 ± 0.0097	0.1660 ± 0.0120	1.476 ± 0.0581	29.10 ± 0.2864
Groups	*P* value for the source of variation			
P–C **vs** N–C	0.3531 (ns)	0.0021 (***)	0.0018 (**)	0.1391 (ns)
P–C **vs** N-MSCs	0.6954 (ns)	0. 0075 (**)	0.8628 (ns)	0.3063 (ns)
P–C vs R-MSCs	0.4136 (ns)	0.0051 (**)	0.0635 (ns)	0.7007 (ns)
P–C vs D-MSCs	0.6219 (ns)	0.0022 (**)	0.8710 (ns)	0.2021 (ns)
P–C vs DR-MSCs	0.9989 (ns)	0.0010 (**)	0.7541 (ns)	0.8277 (ns)

The statistical significance was determined by One-way analysis of variance (ANOVA). Tukey’s multiple comparisons were used to determine the relationship between the variable’s means. N–C: Negative Control (Healthy mice treated with PBS); P–C: Positive Control (Pristane-immunized mice treated with PBS); N-MSCs (Naïve MSCs without any interventions); R-MSCs: MSCs exposed to lactobacillus rhamnosus; D-MSCs: MSCs exposed to lactobacillus delbrueckii; DR-MSCs: MSCs exposed to a mixture of lactobacillus rhamnosus and delbrueckii. Data were presented as Mean ± Standard Error of the Mean (SEM). *P* values of ≤ 0.05 were considered significant. (*P ≤ 0.05, **P ≤ 0.01, ***P ≤ 0.001)

### Infusion of PIL mice with naïve MSCs and Probiotic-trained MSCs attenuates the clinical manifestations of lupus differently

**Fig. 3:**
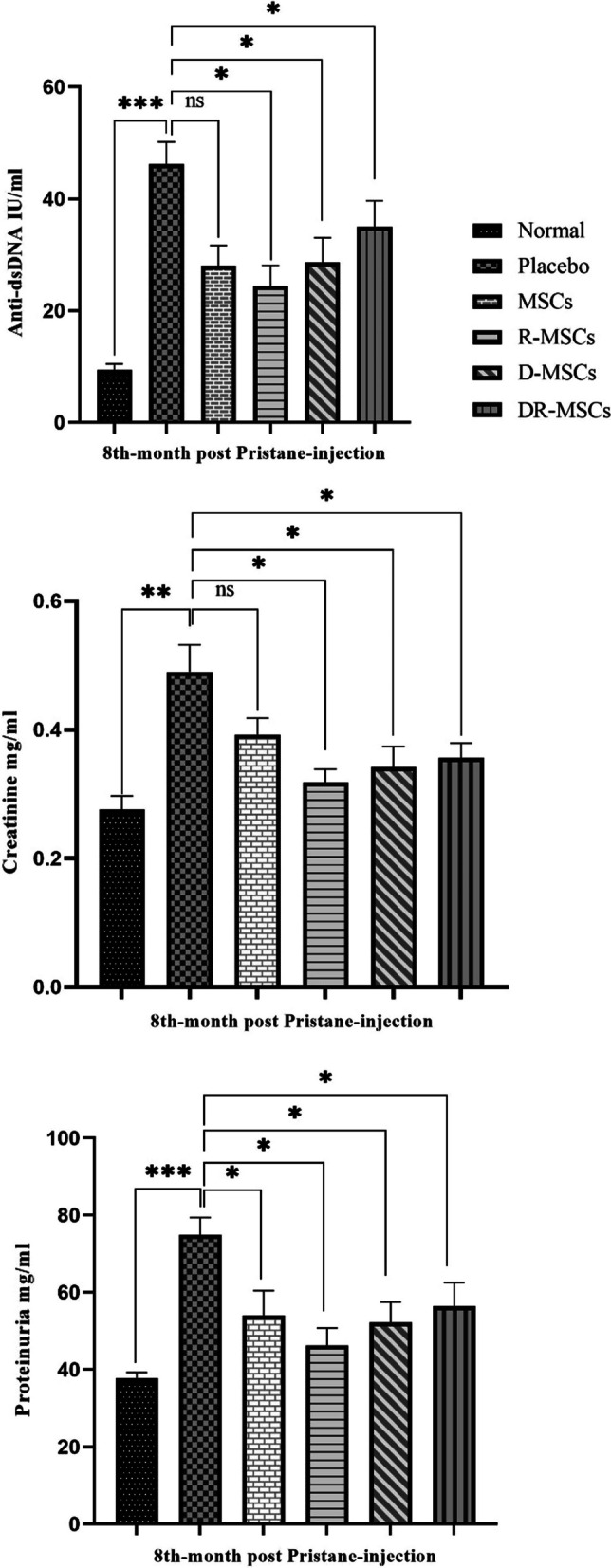
Probiotic coincubation alters MSC immunoregulatory capacity on clinical biomarkers. Levels of anti-dsDNA antibodies and creatinine in sera and urine protein in the P–C group were significantly higher than those in the P–C group. Compared to the P–c group, N-MSCs, R-MSCs, and D-MSCs groups revealed a marked reduction in anti-dsDNA antibodies; DR-MSCs treatment did not induce a similar effect. Moreover, urine protein was statistically significantly lower in mice treated with naïve MSCs or coincubated MSCs compared with the PBS-treated PIL mice model. Although the serum creatinine level of mice treated with naïve MSCs was not significantly different from that in the P–C group, the mean serum creatinine concentration from mice treated with coincubated MSCs was significantly lower than that in the P–C group. N–C: Negative Control (Healthy mice treated with PBS); P–C: Positive Control (Pristane-immunized mice treated with PBS); N-MSCs (Naïve MSCs without any interventions); R-MSCs: MSCs exposed to lactobacillus rhamnosus; D-MSCs: MSCs exposed to lactobacillus delbrueckii; DR-MSCs: MSCs exposed to a mixture of lactobacillus rhamnosus and delbrueckii. Data were presented as Mean ± Standard Error of the Mean (SEM). *P* values of ≤ 0.05 were considered significant. (*P ≤ 0.05, **P ≤ 0.01, ***P ≤ 0.001)

Since association with bacteria might have the capability to activate/deactivate signaling pathways and change MSCs’ behavior [1], the impact of transplanted probiotic-pretreated MSCs on Pristane-induced lupus in mice was evaluated through the analysis of the significant clinical signs (serum level of anti-dsDNA antibodies, creatinine, and urine proteinuria), in comparison with mice that received PBS (P–C group). As shown in Fig. 3A-C, the concentration of serum anti-dsDNA antibodies and creatinine, as well as proteinuria, were significantly higher in the P–C group than those in the N–C group. (P value is presented in Table 2.) As previously described [5], we found transplantation of naïve MSCs significantly downregulated the levels of anti-dsDNA antibodies production, compared with the P–C group in sera collected from mice at 32, and 40 weeks of age. According to the Bonferroni test, transplantation of coincubated MSCs with L. rhamnosus resulted in a significant reduction in anti-dsDNA antibodies compared to the P–C group. (*P* value is presented in Table 2.) Similarly, transplantation of coincubated MSCs with L. delbrueckii significantly decreased the level of this parameter, compared with the P–C group. (P value is presented in Table 2.) Engrafted DR-MSCs tended to decrease the high levels of anti-dsDNA induced by the administration of Pristane, although the difference with the P–C group was not statistically significant. (*P* value is presented in Table 2.) Further, transplantation of coincubated MSCs with L. rhamnosus or L. delbrueckii and/or a mix of probiotics significantly reduced the levels of creatinine in sera, compared with the PIL mice treated with PBS, while, transplantation of naïve MSCs did not show a significant difference to the PIL mice treated with PBS (Fig. 3B). (*P* value is presented in Table 2.) On the other hand, significant inhibition in proteinuria of mice receiving naïve MSCs or MSCs coincubated with L. rhamnosus or L. delbrueckii and/or a mix of probiotics revealed than in PIL mice treated with PBS (Fig. 3C). (*P* value is presented in Table 2.) Meanwhile, the administration of R-MSCs or D-MSCs diminished the level of creatinine and urine proteinuria to the extent that were not significantly different from those measured in the N–C group, however, a less marked decrease was noted in the DR-MSCs group. (Data are presented in Table 2.) Furthermore, transplanted N-MSCs restored the levels of proteinuria within that found in the N–C group, while a less marked decrease was noted in the levels of anti-dsDNA and creatinine. (Data are presented in Table 2.) The administration of N-MSCs, R-MSCs, D-MSCs or DR-MSCs decreased the anti-dsDNA level to a level that was lower than in the P–C group, nevertheless the difference with the N–C group was significant. (Data are presented in Table 2.)

**Table 2:** P values for comparison of anti-dsDNA antibody, creatinine, and proteinuria levels of the experimental groups at the end of the study

Groups	Anti-dsDNA	Proteinuria	Creatinine
P–C vs. N–C P–C vs. N-MSCs P–C vs. R-MSCs P–C vs. D-MSCs P–C vs. DR-MSCs N–C vs. N-MSCs N–C vs. R-MSCs N–C vs. D-MSCs N–C vs. DR-MSCs	0.0006 (***) 0.0236 (*) 0.0155 (*) 0.0202 (*) 0.0650 (ns) 0.0039 (**) 0.0211 (*) 0.0191 (*) 0.0070 (**)	0.0003 (***) 0.0247 (*) 0.0134 (*) 0.0264 (*) 0.0434 (*) 0.0715 (ns) 0.1471 (ns) 0.0573 (ns) 0.0455 (*)	0.0038 (**) 0.1336 (ns) 0.0270 (*) 0.0396 (*) 0.0264 (*) 0.0355 (*) 0.1222 (ns) 0.1506 (ns) 0.0130 (*)

The statistical significance was determined by One-way analysis of variance (ANOVA). Bonferroni’s multiple comparisons were used to determine the relationship between the variable’s means. N–C: Negative Control (Healthy mice treated with PBS); P–C: Positive Control (Pristane-immunized mice treated with PBS); N-MSCs (Naïve MSCs without any interventions); R-MSCs: MSCs exposed to lactobacillus rhamnosus; D-MSCs: MSCs exposed to lactobacillus delbrueckii; DR-MSCs: MSCs exposed to a mixture of lactobacillus rhamnosus and delbrueckii. Data were presented as Mean ± Standard Error of the Mean (SEM). *P* values of ≤ 0.05 were considered significant. (*P ≤ 0.05, **P ≤ 0.01, ***P ≤ 0.001)

### Novel generation of MSCs could manage kidney microstructure

**Fig. 4:**
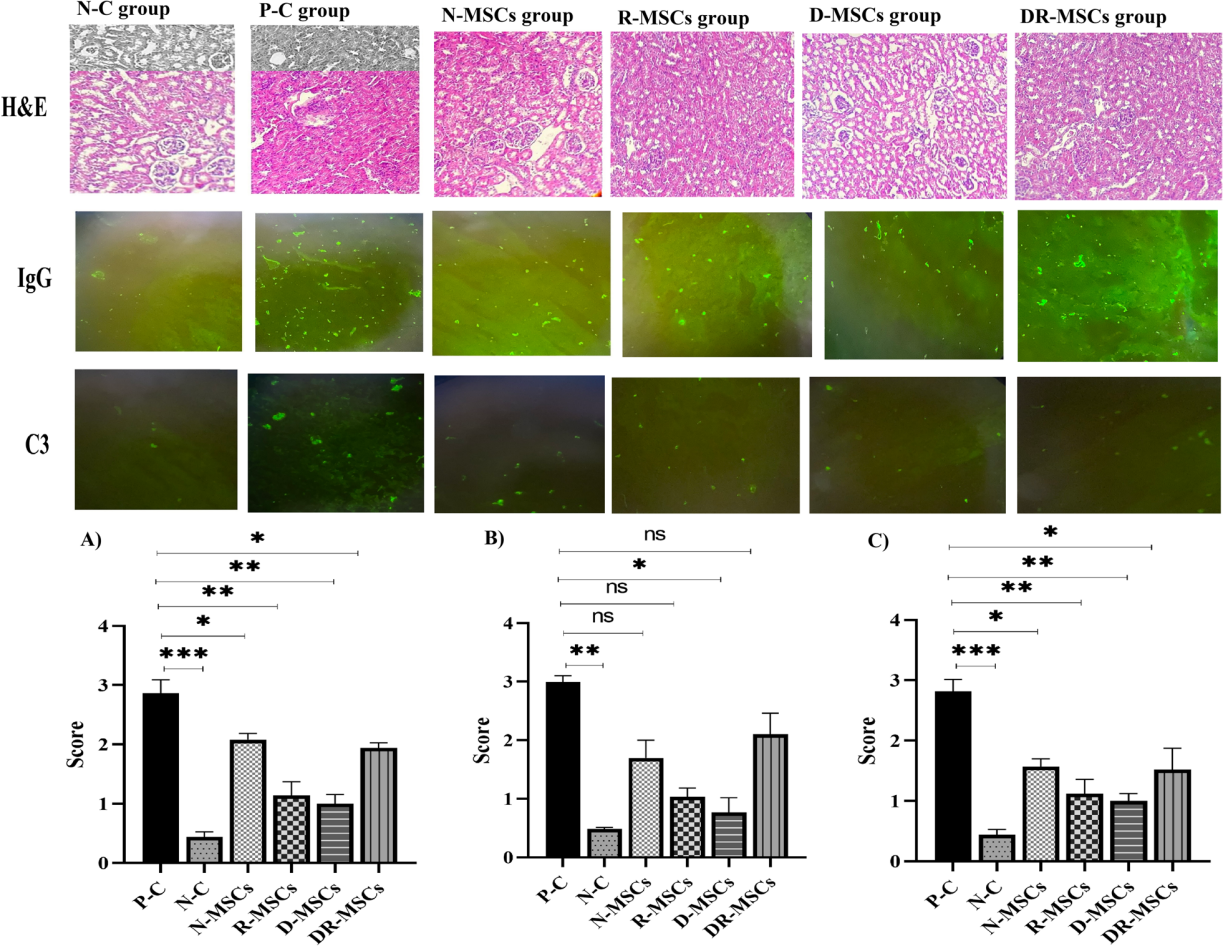
The comparative analysis of therapeutic effects of engrafted naïve MSCs or probiotic-educated MSCs on kidney microstructure. **A**. Hematoxylin and eosin-stained kidney sections of different groups at magnification × 100. Examination of H&E sections of the normal mice (N–C group) showed normal kidney architecture. In naïve MSCs, injection appeared to have a relatively normal structure in some areas, compared with the P–C group. Probiotic-trained-MSCs transplantation improved renal microstructure in the PIL mice model, including reduced basal membrane disorder, mesangial cell overgrowth in glomerular, and infiltration of cells. B and C). By the results of the light microscopic study, immunofluorescence analysis was performed on kidney sections obtained from mice in all experimental groups to evaluate the fluorescence intensity of immune complexes containing IgG (middle panels) or C3 (lower panels) associated with the immune response and inflammation. We found that PIL mice presented a significant increase in fluorescence intensity compared to normal mice. A remarkable reduction in the fluorescence intensity was seen in all MSCs treatments (either naïve MSCs or probiotic-pretreated MSCs) compared to the PIL mice treated with PBS. The administration of MSCs remarkably alleviated inflammation induced by Pristane compared with the PBS-treated mice; a highly significant difference in the parameters was detected in the D-MSCs group compared to other groups. Bonferroni’s multiple comparisons were used to determine the relationship between the variable’s means. N–C: Negative Control (Healthy mice treated with PBS); P–C: Positive Control (Pristane-immunized mice treated with PBS); N-MSCs (Naïve MSCs without any interventions); R-MSCs: MSCs exposed to lactobacillus rhamnosus; D-MSCs: MSCs exposed to lactobacillus delbrueckii; DR-MSCs: MSCs exposed to a mixture of lactobacillus rhamnosus and delbrueckii. Data were presented as Mean ± Standard Error of the Mean (SEM). *P* values of ≤ 0.05 were considered significant. (*P ≤ 0.05, **P ≤ 0.01, ***P ≤ 0.001)

The effects of engrafted MSCs on kidney microstructure were determined by H&E staining and immunofluorescence analysis. Figure 4 shows kidney sections prepared from mice euthanized at the end of the study and stained with H&E to evaluate inflammation. Upon histopathological analysis of the kidney, light microscopic examination showed that normal mice as the N–C group presented normal kidney histology. At the same time, Pristane injection provoked the loss of kidney architecture, including glomerular basal membrane disorder, mesangial cell overgrowth, and mild-to-moderate infiltration of inflammatory cells in the interstitial and surrounding vessels. In contrast, a marked decrease in the infiltrating cells in the kidney of PIL mice was seen after administration of either naïve MSCs or probiotic-educated MSCs, compared to PBS-treated PIL mice. The administration of R-MSCs and D-MSCs almost abrogated these kidney changes. In the group treated with D-MSCs, however, a more pronounced reduction in infiltrating inflammatory cells was observed. Likewise, Mice in the N-MSCs group and mice in the DR-MSCs group exhibited fewer inflammatory cells in the surrounding vessels than the P–C group. Figure 4A shows the comparison of histopathological scores between groups. There was a significant difference between N–C and P–C groups regarding histopathological scores (*P* = 0.0006).

Mice in P–C group obtained a score of 2.892 ± 0.1032, which was significantly reduced to 2.07 ± 0.0477, 1.14 ± 0.1030, 1.01 ± 0.707, and 1.94 ± 0.0400 in treated mice with naïve MSCs (P = 0.0185), rhamnose-pretreated MSCs (*P* = 0.0024), delbrueckii-pretreated MSCs (P = 0.0014), and delbrueckii/rhamnosus-pretreated MSCs (*P* = 0.0150), respectively. The H&E score was significantly higher in N-MSCs, R-MSCs, D-MSCs, and DR-MSCs groups compared to the N–C group (*P* = 0.0004; *P* = 0.0462; *P* = 0.0176 and *p* = 0.0002, by Bonferroni’s tests, respectively) (Fig. 4A). Because previous studies have revealed the close relationship between the precipitation of immune complexes and kidney function, we further investigated how this parameter changed upon transplantation of probiotic-trained MSCs. Figure 4B and C shows pathological sections of the mice kidney that were used for immunofluorescence analysis. PIL mice presented a significant increase in fluorescence intensity of IgG (*P* = 0.0085) and C3 (*P* = 0.0006) compared to normal mice. Precipitation of immune complexes containing IgG and C3 has markedly decreased in the kidney tissue of the R-MSCs and D-MSCs groups when compared with the P–C group. However, the administration of D-MSCs decreased the precipitation of immune complexes to reach a level similar to that in the negative control. We also found that the fluorescence intensity of IgG and C3 was markedly reduced within kidney structure in N-MSCs and DR-MSCs groups, compared with the P–C group. The PBS-treated PIL mice obtained a score of 3.01 ± 0.0577 (for IgG), whereas a significantly reduced score of 1.7 ± 0.1732 in N-MSCs (*P* = 0.2029) 1.03 ± 0.0881 in R-MSCs (*P* = 0.0557), 0.76 ± 0.145 in D-MSCs (P = 0.0233) and 2.1 ± 0.2082 in DR-MSCs (*P* = 0.9999) groups was observed. The IgG score was higher in N-MSCs, R-MSCs, D-MSCs, and DR-MSCs groups compared to the N–C group (P = 0.3452; *P* = 0.5100; *P* = 0.9999 and *P* = 0.2168, by Bonferroni’s tests, respectively) (Fig. 4B). The PBS-treated PIL mice obtained a score of 2.814 ± 0.0900 (for C3) whereas a significantly reduced score of 1.566 ± 0.0587 in N-MSCs (*P* = 0.0112); 1.12 ± 0.1068 in R-MSCs (*P* = 0.0045); 1.01 ± 0.0547 in D-MSCs (*P* = 0.0022) and 1.52 ± 0.1594 in DR-MSCs (*P* = 0.0365) groups was observed. The C3 score was significantly higher in N-MSCs, R-MSCs, D-MSCs, and DR-MSCs groups compared to the N–C group (*P* = 0.0005; *P* = 0.0468; *P* = 0.0110 and *P* = 0.0182, by Bonferroni’s tests, respectively) (Fig. 4C).

### Probiotic-trained MSCs effects on serum cytokines levels in PIL mice model

**Fig. 5:**
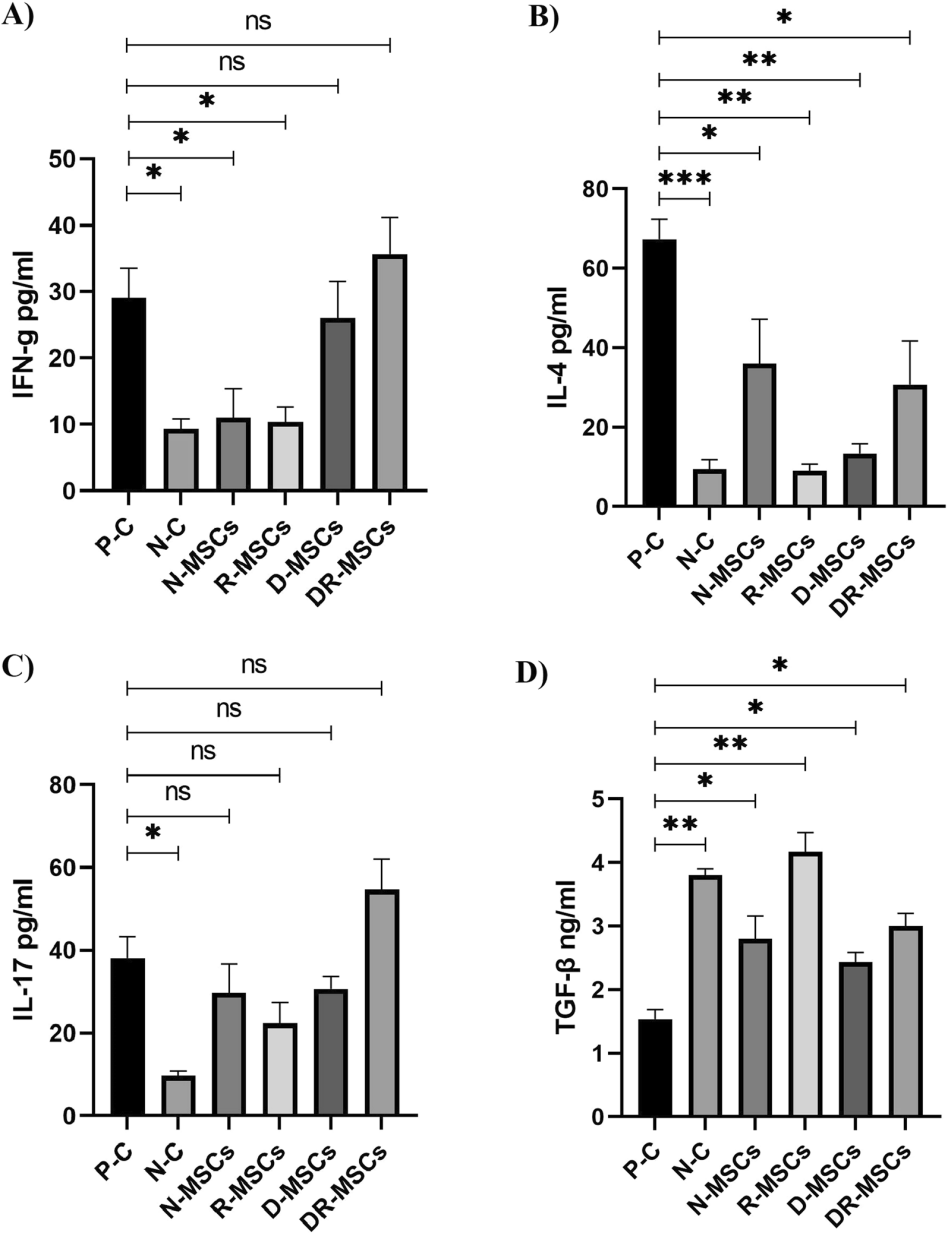
Probiotics showed modulatory effects on MSCs activity. The N-MSCs group reduced the serum levels of IL-4, IL-17, and IFN-ɣ compared to the P–C group. The reduction was found to be significant in the case of IL-4 and IFN-ɣ. Compared to the P–C group, in the R-MSCs group, the reduction in the serum level of the mentioned cytokines had the same pattern as in the N-MSCs group, but the intensity of the diminish was more noticeable. In the group which received L. delbrueckii-pretreated MSCs, a significant reduction was found in the serum level of IL-4, accompanied by a nonsignificant reduction in the IL-17, with no change in the level of IFN-ɣ, when compared to the P–C group. On the other hand, compared with the P–C group, despite the significant decrease in the serum level of IL-4, the DR-MSCs group exhibited stimulant effects for IFN-ɣ and IL-17, as inflammatory inducers, which not verified our hypothesis. Actually, in contrast to MSCs pretreated by L. rhamnosus or L. delbrueckii, a mixed suspension of probiotics provoked MSCs to increase inflammatory cytokines. In addition, TGF-β was significantly found to be enhanced in MSC-treated groups. It was the highest in the sera of the R-MSCs group, while it was the lowest in the sera of the P–C group. These findings revealed that bacteria can have a vital role in modulating MSC–host immunocytes interactions in the systemic immune system. Tukey’s multiple comparisons was used to determine the relationship between the variables means. N–C: Negative Control (Healthy mice treated with PBS); P–C: Positive Control (Pristane-immunized mice treated with PBS); N-MSCs (Naïve MSCs without any interventions); R-MSCs: MSCs exposed to lactobacillus rhamnosus; D-MSCs: MSCs exposed to lactobacillus delbrueckii; DR-MSCs: MSCs exposed to a mixture of lactobacillus rhamnosus and delbrueckii. Data were presented as Mean ± Standard Error of the Mean (SEM). *P* values of ≤ 0.05 were considered significant. (*P ≤ 0.05, **P ≤ 0.01, ***P ≤ 0.001)

To determine if our treatment protocols can modulate pristane-induced cytokines, we measured the serum levels of IFN-ɣ, IL-4, IL-17, and TGF-β in all experimental groups. Naïve MSCs were coincubated with Lactobacillus strains before administration according to the protocol. At the end of the study, blood was collected from mice in all experimental groups for specific cytokines determination and examined using ELISA Kit. The mean levels of IFN-ɣ (*P* = 0.0311), IL-4 (*P* = 0.0009), and IL-17 (*P* = 0.0477) in sera from the P–C group were significantly higher, and that of TGF-β (*P* = 0.0018) was significantly lower than those in the N–C group (Fig. 5A-E). Transplantation of naïve MSCs and MSCs pretreated with L. rhamnosus was able to reduce the serum level of IFN-ɣ significantly (P = 0.0252 and *P* = 0.0344, respectively) and IL-4 (*P* = 0.484 and *P* = 0.0007, respectively; Fig. 5A and B), compared to treatment of the PIL mouse model with PBS. As can be seen in Fig. 5A and B, transplantation of D-MSCs did not cause a significant change in the serum level of IFN-ɣ (P = 0.9708) despite a significant decrease in the serum level of IL-4 (*P* = 0.0001), compared with the PIL mice model treated with PBS. On the other hand, compared to the P–C group, transplantation of MSCs pretreated with a mix of L. rhamnosus and L. delbrueckii significantly downregulated the level of IL-4 (*P* = 0.0405) in sera, although the serum level of IFN-ɣ (*P* = 0.8335) increased, however, this increase failed to reach statistical significance (Fig. 5D). Notably, administration of probiotic-pretreated MSCs with strain-specific displayed different cytokine patterns, and none of the studied groups mirrored each other’s effect. While R-MSCs significantly reduced IFN-ɣ, it was only borderline decreased by D-MSCs treatment; also increase was noted in DR-MSCs. IL-17 was equivalently decreased in the PIL mice model treated with N-MSC (*P* = 0.7162) or D-MSCs (*P* = 0.5915), while a further reduction was observed in mice treated with R-MSCs (*P* = 0.2906), compared with the P–C group. However, IL-17 level in sera was increased in mice treated with DR-MSCs (*P* = 0.3059) compared to the P–C group. The serum level of TGF-β was significantly higher in mice treated with N-MSCs (*P* = 0.0385) compared with the P–C group. As can be seen in Fig. 5D, a less significant increase was noted in mice treated with D-MSCs (*P* = 0.0147), while R-MSCs (*P* = 0.0082) and DR-MSCs (*P* = 0.0131) transplantation induced a marked increase in the level of TGF-β, compared with the P–C group. Our results offer novel insights into the effect of probiotics on modulating the MSCs’ immune plasticity, which influences the differentiation of pro- or anti-inflammatory cytokines in the lupus microenvironment.

### Naïve MSCs and Probiotic-trained MSCs reduce Th cell populations in splenocytes, but potency was varied

**Fig. 6:**
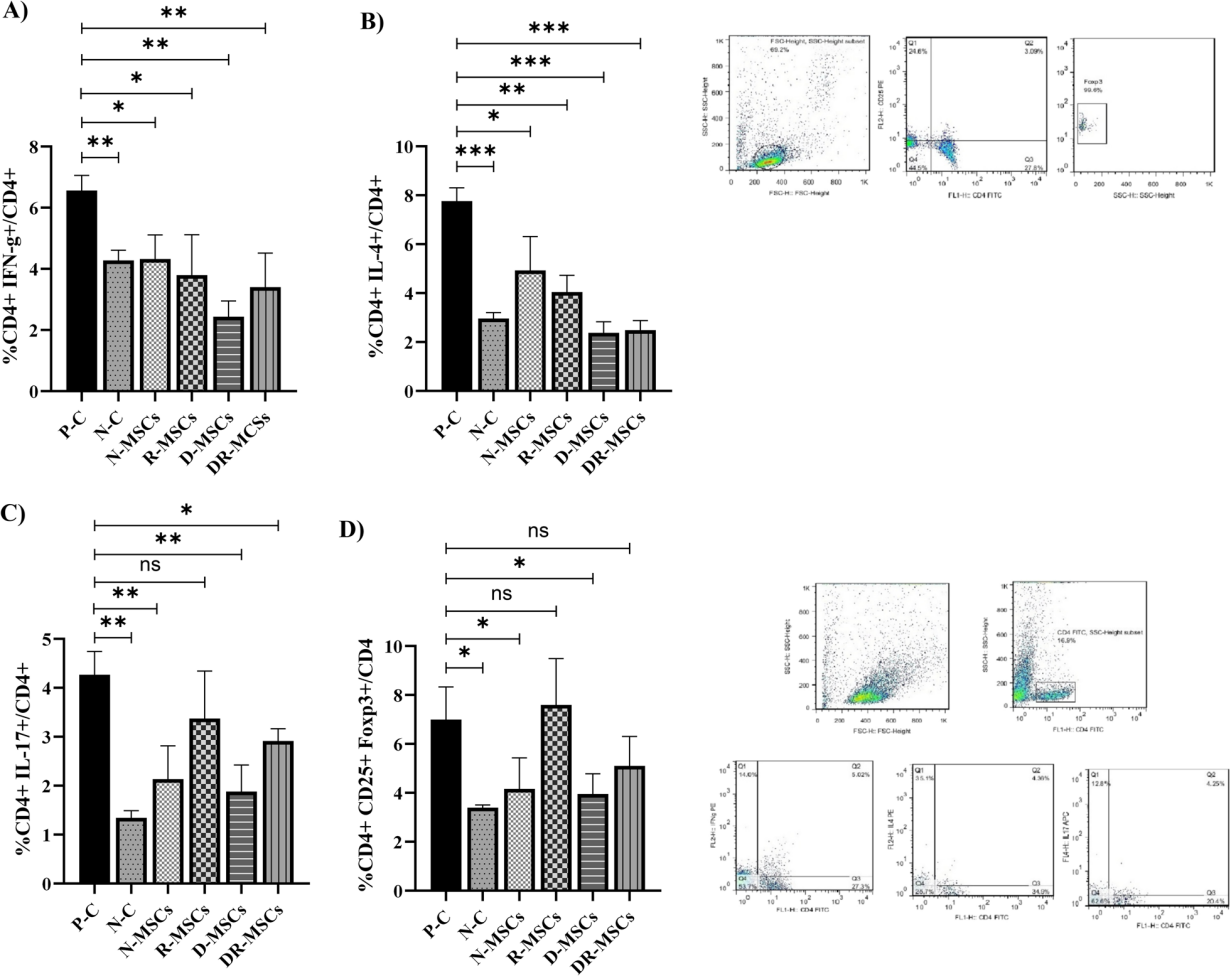
Flow cytometric analysis to evaluate the effect of engrafted MSCs on the frequency of splenocytes. A representative gating scheme and representative dot plots are also presented. Th1, Th2, Th17, and Treg percentages were significantly higher in the P–C group than in the N–C group. The population of Th1 and Th2 in all MSCs treated groups was significantly lower than in the P–C group (A and B). Transplantation of naïve MSCs and pre-exposure MSCs to L. delbrueckii significantly decreased the percentage of Treg and Th17 cells compared with the P–C group. However, there was no significant difference between the percentage of Th17 and Treg cells of mice treated with pre-exposure MSCs to L. rhamnosus with those of the P–C group. Furthermore, the percentage of Th17 cells in the DR-MSCs group was significantly reduced. However, no significant differences were observed regarding the percentage of Treg cells between the DR-MSCs and P–C groups. These findings support the hypothesis that bacteria can interact with MSCs and educate MSCs with potent immunosuppressive and immunomodulatory properties. Interestingly, a mix of bacteria did not have a similar effect as R-MSCs or D-MSCs. Tukey’s multiple comparisons were used to determine the relationship between the variable’s means. N–C: Negative Control (Healthy mice treated with PBS); P–C: Positive Control (Pristane-immunized mice treated with PBS); N-MSCs (Naïve MSCs without any interventions); R-MSCs: MSCs exposed to lactobacillus rhamnosus; D-MSCs: MSCs exposed to lactobacillus delbrueckii; DR-MSCs: MSCs exposed to a mixture of lactobacillus rhamnosus and delbrueckii. Data were presented as Mean ± Standard Error of the Mean (SEM). *P* values of ≤ 0.05 were considered significant. (*P ≤ 0.05, **P ≤ 0.01, ***P ≤ 0.001)

To reveal which subpopulation of CD4^+^ cells could be modulated in the lupus microenvironment by probiotic-trained MSCs, we used immunostaining and flow cytometry to investigate and compare frequency distribution in Th1, Th2, Th17, and Treg between six experimental groups (Fig. 6A-D). In splenocytes from the P–C group, the proportion of Th1 (*P* = 0.0017) and Th2 (*P* = 0.0001) was significantly higher than those in the N–C group. Mice treated with MSCs significantly downregulated the percentage of Th1 (Fig. 6A) and Th2 (Fig. 6B) cell subpopulations in a naïve state (*P* = 0.0401 and *P* = 0.0481, respectively), pretreated with L. rhamnosus (*P* = 0.0189 and *P* = 0.0032, respectively), or L. delbrueckii (*P* = 0.0013 and *P* = 0.0009, respectively), and/or a mix of probiotics (*P* = 0.0062 and *P* = 0.0006, respectively), compared with the P–C group. Meanwhile, D-MSCs and DR-MSCs groups decreased the percentage of Th1 and Th2 cell subpopulations to a greater extent than N-MSCs and R-MSCs groups.

The proportion of CD4^+^ IL-17^+^ (Fig. 6C) and CD4^+^ CD25^+^ FOXP3^+^ (Fig. 6D) cells in the P–C group (*P* = 0.0018 and P = 0.0251, respectively) was significantly higher than those in the N–C group. As displayed in Fig. 6C, transplantation of naïve MSCs (*P* = 0.0098), pretreated MSCs with L. delbrueckii (*P* = 0.0059), and pretreated MSCs with a mix of L. delbrueckii and L. rhamnosus (*P* = 0.0301) significantly downregulated the percentage of Th17 cell subpopulation, compared to the P–C group. However, N-MSCs and D-MSCs groups induced more Th17 reduction than the DR-MSCs group (two stars against one star). A similar result (Fig. 6C) was seen in the R-MSCs group (*P* = 0.2091 compared with the P–C group, although this reduction in the Th17 cell subpopulation failed to reach statistical significance.

The N-MSCs and D-MSCs groups downregulated the percentage of Treg cell populations (*P* = 0.0450 and *P* = 0.0359 values, respectively; Fig. 6D) compared with the P–C group. In group DR-MSCs, mice also reduced the increased population of Treg cells induced by the lupus microenvironment from (6.992 ± 0.5970%) in the P–C group to (5.100 ± 0.5375%), although this reduction failed to reach statistical significance (*P* = 0.2573; Fig. 6D). While the D-MSCs group reduced as much Treg cell percentage as the N-MSCs group, mice that received MSCs pretreated with L. rhamnosus upregulated Treg cell subpopulation, compared to the P–C group, albeit this difference failed to reach statistical significance (*P* = 0.9936) (Fig. 6D).

## Discussion

The efficacy of cell therapies has long been discussed in several autoinflammatory disorders, yet no efficient treatment has been established for treating all cases of SLE [68–71]. According to data, one potential mechanism that can possibly explain the development and pathogenesis of SLE is the alteration of MSCs in terms of their quantity, characteristics, and functionality. The broad evidence has demonstrated that the majority of immune cells, including MSCs, possess a dualistic nature, wherein they are capable of adopting either tolerogenic or inflammatory phenotypes depending on the surrounding milieu [3]. Hence, targeting MSCs and manipulating them may offer significant clinical benefits in the treatment outcome of SLE. Previous studies have shown improvements after allogenic MSC-based therapy in both mouse models of SLE and patients (reviewed in [3, 14]). MSCs are the focus of extensive investigation as a natural biotreatment due to their unique features, including lack of immune activity, inherent ability to fight inflammation, and promote immunological tolerance [64, 72]. At present, there is limited information concerning the manipulation of MSCs for SLE treatment. In addition, a wide variety of pharmaceutical and biological agents employed to manipulate MSCs give rise to the uncertain phenotypic and functional characteristics of MSCs [73]. Existing studies have shown that probiotics have a close relationship with different cells in various organs at the systemic level [58]. This includes the intricate interplay between the intestine and kidney [74–76], the liver and intestine [77–79], as well as the lung and intestine [80–82]. Probiotics generate a variety of enzymes and molecules, which possess the potential to control signaling pathways, modulate the expression of specific genes, and regulate a multitude of targets in order to manifest probiotic effects [50, 54, 56]. Intriguingly, Lactobacillus delbrueckii and L. rhamnosus have been capable of inducing the tolerogenic phenotype of certain immune cells from SLE patients and lupus mouse models (review in [47, 73, 83]). In addition, the concept has emerged that abnormalities in MSCs significantly affect the progression and complications of autoimmune diseases such as SLE. Intriguingly, recovering the function of deficient MSCs by Lactobacillus supplementation has been reported [64, 84]. The potential for interaction between MSCs, microbiota, and the immune system has fueled research into interventions to modify the MSCs to treat immune-mediated disease. Recently, it has been found that signals from environmental factors train naïve MSCs to become educated MSCs that act differently [85]. Little is known about how bacteria and bacterial components interact with MSCs and how contact with bacteria affects the immunoregulatory potential of MSCs. Consequently, it is of great significance to explore the outcome of therapeutic protocol probiotic-educated MSCs (pre-exposure MSCs to L. rhamnosus or L. delbrueckii or a mix of them (since most of the probiotics used in studies are mixed formulation [86])) on clinical, serological, and immunological abnormalities in an animal model of lupus disease induced by Pristane. To the best of our knowledge, this study may be the first to describe the effects of beneficial bacteria on the function of MSCs in vivo conditions by investigating the therapeutic effects of bacteria-primed MSCs on SLE-like symptoms. Obtained data revealed significant changes in MSCs’ behavior that were bacteria-dependent. We suggest that in the context of lupus in clinical studies, considering the inflammation conditions, MSCs pretreated with the strains of L. rhamnosus or L. delbrueckii may show better treatment outcomes than N-MSCs. We preferred to study L. rhamnosus and L. delbrueckii since these strains were reported to regulate innate and adaptive immune systems and are well known for their antioxidant, anti-inflammatory, and anticancer properties [12, 87]. Transplantation of pretreated MSCs with probiotics used in this study provided the advantages of distinct immunoregulatory ability in different Lactobacillus strains toward MSCs.

Our Pristane-induced lupus mouse model developed expanded lipogranuloma lesions; in contrast, mice treated with infusion of probiotic-pretreated MSCs experienced significantly fewer such inflammation lesions on gross at 40 weeks of age. This effect coincided with a significant reduction in spleen weight, suggesting that the probiotic-pretreated MSCs can reduce lymphoid hyperplasia. In contrast to probiotic-pretreated MSCs, N-MSCs treatment did not reduce lipogranuloma lesions. It is worth noting that crosstalk between MSCs and the bacteria have altered the migration ability and transcription of vital immunomodulatory genes, which profoundly affected MSCs’ function [1]. Meanwhile, apoptotic cells-treated MSC has been reported to express chemokine receptors that could guide the migration of MSCs [88]. Interestingly, probiotic-pretreated MSCs could physically access the lipogranuloma lesions as a secondary lymphoid organ and reduce them significantly, suggesting that probiotic-pretreated MSCs might also be beneficial for treating other autoinflammatory disorders [89].

Regarding biodistribution, evidence of many MSCs was found in the liver and spleen when administered intravenously. Compared to the P–C group, administration of naïve MSCs significantly reduced spleen weight; however, pretreatment with probiotics augmented the inhibitory effect that the MSCs had on spleen weight compared with naïve MSCs. It agrees with Silva and colleague findings, who suggested that increased engrafted MSCs in the spleen suppress T cell proliferation, allowing the host to avoid an excessive immune response that may also cause damage [90]. Meanwhile, the results of a study by Santos Rocha et al. showed that the administration of lactobacilli modulates spleen, lymph nodes, and systemic immune responses in animal models of experimental colitis [91]. Likewise, liver weight, which was increased in PBS-treated PIL mice, compared to normal mice, was reduced in mice treated with naïve MSCs or probiotic-pretreated MSCs. Sun et al. reported that hepatomegaly indicates liver dysfunction in SLE mice, while the injection of MSCs can reverse the histological changes associated with SLE in the liver [92]. Oxidative free radicals are unequivocally associated with various inflammatory diseases and act as cellular signals modifying the surrounding microenvironment [93, 94]. Recent in vivo and in vitro studies have shown that MSCs have antioxidant capacity, and intravenous injection effectively modulates oxidative stress in tissues such as the kidney and liver [95]. These suggested effects were secondary to the immunomodulation of pro-inflammatory signaling, including a reduction in inflammatory cytokines IFN-ɣ and IL-4 [96, 97]. Interestingly, investigators provided evidence that MSCs treatment could improve liver function, alleviate hepatic inflammation, and contribute to liver fibrosis regression [98–100]. However, it has been documented that MSC-based therapy improves liver function during the first six months after administration [101]. Evidence has shown that probiotics have successfully reduced oxidative stress in liver and kidney tissue due to the existence of the gut-liver and gut-kidney axis [86, 102, 103].

Since the serum level of anti-dsDNA antibodies plays a vital role in lupus nephritis, and urine protein and serum creatinine levels are essential biomarkers in the interpretation of kidney function, we measured and compared the mean levels of these parameters before and after treatment strategies. Compared with the P–C group, lower levels of these parameters (serum levels of anti-dsDNA antibodies, creatinine as well, as urinary protein) were determined in the N-MSCs group, as confirmed by Sun et al. [92], Chang et al. [104], Dang et al. [105], and Wang et al. [106] who reported that transplanted naïve MSCs can be effective in limiting lupus nephritis. Probiotic pretreated MSCs treatment also decreased the level of anti-dsDNA antibodies; however, in contrast to R-MSCs and D-MSCs groups, the reduction was not statistically significant in the DR-MSCs group than in the P–C group. The urine protein and serum creatinine concentrations were significantly lower in the PIL mice model treated with probiotic-pretreated MSCs than in PBS-treated ones with the lower quantity of these parameters (urine protein and serum creatinine) in mice receiving R-MSCs. Notably, proteinuria and serum creatinine concentrations were lower in the N–C group than in other groups. However, differences were not significant than those in the R-MSCs and D-MSCs groups. Moreover, no statistically significant difference was observed in proteinuria between the N–C and N-MSCs groups, although this condition was not observed in serum creatinine levels. In general, transplanted MSCs pretreated with L. rhamnosus or L. delbrueckii showed more favorable serum creatinine and urine protein results than the N-MSCs group, inferring that they can improve the microstructure of the kidneys. Recently, therapeutic modulation of probiotics has been suggested to be one of the tools for reducing inflammation and delaying the progression of kidney failure [86]. Substantial evidence has shown that using probiotics dramatically affects the serum level of anti-dsDNA antibodies, creatinine, and urine protein attenuation [107–110]. Aggregation ability and adhesion to host tissues have been considered critical criteria among the bacterial strains used as probiotics. Strikingly, variation in the total adhesion, externalization, and internalization abilities among individual strains of Lactobacillus is excellent [46]. The study conducted by Mendia et al. exhibited that L. rhamnosus had a strong adhesive affinity on MSCs [11]. Bacterial adherence and invasion do not alter MSC viability or proliferation [1]. Moreover, Kol et al. microscopically confirmed that probiotics, Lactobacillus strains, appeared and degraded in the cytoplasmic matrix of MSCs seven h post-coincubation [1]. Probiotics, on the other hand, can regulate MSC differentiation and function. Tso and coauthors demonstrated that MSCs can phagocytose apoptotic cells, which modulates MSC’s differentiation and function [88]. In addition to phagocytosis, interactions are an essential function in activating or deactivating cells and/or acquiring any abilities related to new properties and outcomes. De Marco et al. provided evidence that certain probiotic supernatants (probiotic metabolites) can differently modulate biomarkers expression of immune cells in a dose-dependent manner as a peculiar adjuvant in anti-inflammatory therapy [111]. As a consequence of interaction, the preference would orient the cell response according to the probiotic strain since probiotics can modify and modulate the immune cell properties [11, 112]. Incubation is the oldest and most commonly used method to load cargo into cells [113]. In the present study, MSCs were incubated with probiotics under appropriate culture conditions for 48 h, and MSCs may phagocytose probiotics. However, the mechanism of that is far from being fully understood. We suggest that L. rhamnosus and L. delbrueckii show potential for an immunophenotype shift or a clinical probiotic effect in unmatured MSCs. Moreover, it may not be surprising if the gene expression state in MSCs will be according to the microbial challenge confronted [66] since the influence of probiotics on the signalization of cell receptors has been reported [84, 102].

By the results mentioned above, further histological analysis with H&E staining revealed the presence of inflammatory cells and microstructure changes in the kidney of PIL mice. Numerous studies have uncovered that stromal cells of mesenchymal origin, including MSCs, promote tissue regeneration through their direct interactions with different types of immune cells, tissue-specific progenitor cells, and major constituents of the tissue microenvironment [95, 114–118]. In a histopathological examination of the kidney, PIL mice treated with PBS showed mild-to-moderate or moderate glomerulonephritis and infiltration of inflammatory cells in the surrounding vessels. However, almost all MSC-treated mice showed relatively benign lesions and only mild infiltration of inflammatory cells. MSCs administration is reported to ameliorate renal parameters regarding dysfunction and morphological abnormalities [61]. We previously reported that N-MSCs attenuate Pristane-induced lupus in BALB/c mice [5]. In the present study, we observed an even stronger anti-lupus effect of probiotic-trained MSCs as we did perform experiments using BALB/c mice in parallel in the same laboratory. Evidence provided that probiotic administration could treat and prevent inflammation attributed to crosstalk between bacteria and the immune and non-immune cells [119, 120]. Similar results have been drawn from the other studies on glomerulonephritis, which were improved significantly with prebiotics and probiotics [87, 110, 121–123]. While D-MSCs abolished the infiltration of cells and significantly attenuated lupus nephritis, at the histological level, the administration of R-MSCs or D-MSCs strongly diminished the tissue damage induced by Pristane, demonstrating their anti-inflammatory effects and regeneration capacity. It could be suggested that the probiotic-trained MSCs might markedly reduce the recruitment of inflammatory cells to inflamed tissues (especially in the case of D-MSCs) [124]. In addition, there was no significant difference regarding the infiltration of cells between N-MSCs, R-MSCs, and DR-MSCs treatment groups; however, potent reduction in the D-MSCs group, illustrating that immunomodulation by R-MSCs in advance has a much higher beneficial effect in reducing inflammation. Several studies have shown that MSCs can induce tolerance by direct effects and via mobilization of suppressor cell populations (reviewed in [14]). In explaining the higher population of infiltrating cells in mice treated with rhamnosus-pretreated MSC than D-MSCs, it can be suggested that R-MSCs may promote the mobilization of immune regulatory cells to the kidney, after that, they act in ameliorating tissue damage [90]. In agreement with our findings, crosstalk between probiotic-trained MSCs and suppressor cells may explain the changed immunomodulatory potential observed in vivo [90]. Likewise, it has been proposed that the beneficial effects exerted by R-MSCs can be associated with a downregulation of both Th1-Th17-driven autoimmune and inflammatory responses [120]. Our results demonstrate that pretreatment by probiotics might potentiate the immunomodulatory effects of MSCs in our model of lupus disease. Despite clear signs of inflammation in the P–C group by the end of the experiment, engrafted D-MSCs or R-MSCs severely affected the infiltration of cells, resulting in a highly significant proteinuria reduction. To examine the shift of the structure in terms of functional activity, we calculated the precipitation of the immune complex containing IgG or C3 between the groups. The fluorescence intensity of C3 deposition in all MSCs treatment groups was significantly lower than in the P–C group. As shown in Fig. 4, the probiotic-trained MSCs treatment groups had a stronger effect in reshaping the structure and improving kidney function than naïve MSCs. The administration of R-MSCs or D-MSCs significantly reduced the precipitation of immune complexes at the end of this experiment. However, the difference between the N-MSCs and DR-MSCs groups was not remarkable. The results above revealed that engrafted MSCs reduce the number of inflammatory cells and the precipitation of immune complexes. These are important mediators associated with clinical symptoms of lupus nephritis, as shown in the mouse model and human disease [125–131]. This reduction in immune complexes correlated with a decrease in proteinuria, as we observed a strong tendency to restore proteinuria of R-MSCs and D-MSCs groups. In the current study, urine protein and creatinine concentrations were near the reference range in R-MSCs and D-MSCs treated mice than in the P–C group. These findings indicate that MSC-associated suppression of autoantibodies may progressively contribute to ameliorating multiorgan dysfunction in PIL mice. Notably, the MSCs effect on lymphocytes B cell appears to occur not only by the modulation of T helper lymphocyte activity but also by direct inhibitory mechanisms by MSC in B lymphocyte activation [61]. These findings are reminiscent of those obtained from ELISA and flow cytometry. R-MSCs might mainly attenuate nephritis symptoms by expanding Tregs subtypes, restoring TGF-β levels to that found in normal mice. At the same time, the anti-nephritis effect of D-MSCs might be mainly correlated with the augmentation of the immunosuppressive effect of naïve MSCs. All in all, while bacteria-MSC coincubation did not induce a harmful phenotype shift, specifically, the final biologic effect depends upon the overall cytokine milieu and the cellular components within the niche, impacting the fate of MSC-plasticity.

To examine whether MSC-exposed to tolerogenic probiotics can modify systemic disease-associated parameters differently than in naïve MSCs, we also compared the serum levels of specific lupus disease-associated cytokines between experimental groups. High levels of IL-4, IFN-ɣ, and IL-17 were determined in the P–C group, as corroborated by researchers in lupus conditions, who reported that high-level expression of IFN-ɣ, IL-17 in lupus-like disease could contribute to the tissue damage [132–135]. As reported previously [5], compared with the PIL mice treated with PBS, naïve MSCs treatment without any pretreated, along with the borderline reduction in IL-17, significantly reduced IL-4 and IFN-ɣ in sera. However, these parameters were found to be reduced to a greater extent in the group that received L. rhamnosus-pretreated MSCs. Notably, despite a significant decrease in IL-4 serum level, variation in IFN-ɣ level was synchronized with altering in IL-17 level. At the same time, injection of R-MSCs reduced them, but D-MSCs did not markedly change them (mild decrease). On the other hand, despite a significant decrease in the serum level of IL-4, pretreated MSCs with a mix of probiotic strains (DR-MSCs) exhibited a stimulant effect for IL-17 and IFN-ɣ as inflammatory inducers, which was inconsistent with our hypothesis. High levels of TGF-β were determined in the MSC-treated mice, consistent with other literature [136–138]. Moreover, it was noted that the R-MSCs group had the highest mean value of TGF-β followed by the DR-MSCs group, then N-MSCs and D-MSCs, and then the P–C group. It is possible that MSC-derived trophic and immunomodulatory mediators with systemic repercussions, such as TGF-β, as a pleiotropic cytokine, replace missing microenvironmental signals [72, 114, 139]. While current studies have screened the therapeutic effects of engrafted MSCs on cytokines secretion in autoinflammatory diseases such as SLE, substantial evidence has shown that using probiotics in inflammatory disease dramatically affects inflammatory cytokines attenuation [140–142]. On the other hand, low levels of IFN-ɣ and IL-17 were determined in the splenocytes coculture probiotics supernatant, as reported by Mardani et al. in an animal model of lupus. One of the characteristics of some bacteria is that it regulates the immune system, whereas they can also contribute to an inflammatory milieu under the influence of circumstances [143, 144]. Parallel, it has been known that other probiotics stimulate and aggravate Th1 immune responses and their cytokines [119]. Notably, Salehipour et al.’s study showed that mixed bacteria had different results than when these probiotic strains were used separately [145]. Another scientist, Kwon et al., showed that while using a mixture of certain probiotics could improve experimental autoimmune encephalomyelitis (EAE), a combination of other probiotics could not alleviate the clinical symptoms of EAE [146]. These results suggested that the new condition might activate or deactivate the expression of any genes (making epigenetic changes) responsible for the suppressive effects. Recent discoveries have also shown that a metastable cell activation state with exclusive gene expression and distinct functional programs may be established through quorum sensing signals in their microenvironment [66]. However, the molecular mechanisms involved in these interactions remain to be attained. In the groups that received L. rhamnosus or L. delbrueckii-pretreated MSCs, a significant reduction in the level of IL-4 without any stimulation in inflammatory cytokines may be suggested as a philosophical investigation in the treatment of allergy diseases. Also, obtained results revealed that the effectiveness of the fight inflammation by R-MSCs was significantly increased than in N-MSCs. R-MSCs were proven to reduce clinical/serological signs of lupus more potently and exhibited a higher immunoregulation ability than N-MSCs. Since enhanced the number of Th1 and Th17 cells is a marker of active SLE, our finding that R-MSCs infusion reduced the serum level of their master cytokines (IFN-ɣ and IL-17) further supports our conclusion R-MSCs combat the increase of inflammation in vivo more effectiveness than the D-MSCs. However, it must be noted that these results were obtained in the default state of the induced lupus model.

We also screened the effects of engrafted pretreated MSCs on the percentage of splenocytes. In the study using the PIL mice model, the population of Th1, Th2, Th17, and Treg cells was significantly higher in the P–C group than in the N–C group. Concerning the percentage of Th1 and Th2 cells, all MSC-treated groups tested in this study displayed significantly fewer populations than the P–C group. MSCs have shown the ability to regulate the balance of Th1/Th2, downregulating Th2-mediated immune responses and IFN-γ [72, 90], as it did in the probiotic-pretreated MSCs treated groups. Moreover, systemic infusion of MSCs significantly decreased the percentage of Th17 cells in the PIL mice model, except in the R-MSCs group.

Furthermore, in the results of the splenocytes from PIL mice, treatment with N-MSCs and D-MSCs led to a significant reduction in the Treg cell subpopulation. However, R-MSCs and DR-MSCs treatment did not significantly change. Researchers reported that MSCs inhibit T cell proliferation by secreting various soluble mediators, direct cell–cell contact, and indirect mechanism. Other studies have shown that probiotics strikingly enhance the levels of TGF-β. Previous reports showed TGF-β to be critical for the Th cell’s balance which is essential in immune homeostasis, inflammation, or tolerance [147]. How TGF-β acts as a suppressive or inflammatory agent in the pathogenesis of SLE is unclear. However, a strong correlation has been reported between TGF-β and some SLE parameters, such as the percentage of TCD4 + IL-17^+^ Foxp3^+^ cells and TCD4^+^ CD25^+^ Foxp3^+^ cells, by targeting the transcription factor RORγ and FOXP3. According to data, it may be suggested that in groups treated with MSCs, there was a strong correlation between levels of TGF-β and Treg cell percentage. Several groups have found that TGF-β is required for the Th17/Treg cell’s balance, raising this suggestion of immunophenotyping shift. Litman et al. reported that Treg generation depends on TGF-receptor signaling in two steps or hits. In contrast to the first, which leads to inducible Foxp3 gene silencing, the latter leads to increased TCD4^+^ Foxp3^+^cells to suppress immune inflammation [66]. Along with this study, other studies have mentioned the dual function of TGF-β, depending on its concentration, on the differentiation of Th cells toward the Th17 and or Treg phenotype [147–149]. On the other hand, Rezalotfi et al. suggested that the plasticity of Th17/Treg cells to acquire inflammatory (TGF-β and IL-6 in the case of Th17) and/or suppressive (TGF-β and IL-2 for Treg cells) phenotypes depending on their environmental cues [150]. There is evidence that loss of high FoxP3 expression results in the capacity to become IL-17-secreting cells under certain inflammatory conditions due to the sensitivity of transcription factors’ expression to environmental signals [151]. Therefore, paying attention to the cytokine microenvironment and immunological context present in lupus (high levels of IL-6 and TNF-α) can probably partially justify the results observed in this study. Moreover, subpopulation-cell analysis in Treg and Th17 cells is needed to inform the delicate balance between inflammatory and suppressive Th cell lineages in the lupus microenvironment, which could lead to solid foundations for developing novel effective biological treatments. It is not far from the mind that condition media can activate/deactivate expressed genes on MSCs, and then MSCs can act differently on different cells.

Although the mechanism is still unclear, reports suggest that some strains of probiotic bacteria are closely associated with induced dedifferentiation of immune cells [44, 152]. Notably, changes in cytokine profile induced by probiotics may be probiotic strain- or site-specific [119, 152]. Our results showed that pretreated MSCs with L. rhamnosus boost TGF-β production while enhancing Treg cell subpopulation. Therefore, TGF-β induction by L. rhamnosus and its effect on Treg rehabilitation through shift immunophenotype is probably one of the mechanisms that apply to boost the immunoregulatory properties of MSCs. However, it conclusively needs to be determined. Probably, alteration in inhibitory or permissive histone modifications in target genes (such as repressing Pax5 results in dedifferentiation of B cells into T cells or expressing a hypomorphic Foxp3 allele results in dedifferentiation of Treg cells into IL-4-producing cells) by the bacterial products [66] can lead to a shift in the phenotype of MSCs, associated with a novel outcome. These results revealed that L. rhamnosus and L. delbrueckii significantly changed the ability of MSCs to regulate the population and maintain the balance of Th1, Th2, Th17, and Treg cells. It must be noted that a significant decrease in TGF-β levels in peripheral blood from SLE patients was also reported. Thus, an increase in TGF-β level by naïve MSCs or probiotic-pretreated MSCs treatment might reduce the breakdown of Th cell balance and decrease the production of pro-inflammatory cytokines. Importantly, alteration in the expression, production, and secretion of other factors of probiotic-pretreated MSCs, which are thought to be involved in the therapeutic mechanism, is not out of mind and needs more extensive studies.

SLE has been considered a disease in which Th2 cytokines, such as IL-4, predominate [153–155]; however, among SLE patients with moderate to severe lupus nephritis, Th1-dominant immune responses [156, 157]. Two stages of T cell activation and cytokine secretion in SLE have been suggested [158]. Hegazy et al. have addressed that depending on microenvironment component signals during the immune response, Th2 cells can be induced to express T-bet and secrete IFN-ɣ since reprogramming of Th1/Th2 cell effector functions may be critical for host defense [159]. Also, it was argued that in response to pro-inflammatory cytokines such as IL-12, Treg cells can produce IFN-γ [160]. Therefore, although FOXP3 expression is a prerequisite for developing Treg cells, not all FOXP3-expressing TCD4^+^ cells appear to be Treg cells, as many Th cells have shown de novo FoxP3 expression transiently under inducible cytokine milieu in a Treg fate-independent manner. Further, there is evidence that depending on their microenvironment, Th17 cells can accept either pro or anti-inflammatory (Th1/Treg) phenotypes through epigenetic mechanisms [66, 150]. In addition, it was suggested that in the Th1-Th17-Treg axis, despite the mutual transdifferentiating Th17/Treg, there is an inability of committed Th1 to convert to Treg [150]. Th17 cells capable of producing both IL-17 and IFN-γ (termed as Th17/Th1 cells) have been reported by Annunziato and colleagues [120]. Increasing, albeit confounding, evidence points to the different cell origins responsible for this discrepancy, adding further complexity to metastable state in Th cells lineage such as Th1-like Tregs and Th1-like Th17 [151]. Overall, positive feedback loops in the induction of Th1 and Th17 cells indicate that the control of these subpopulations is more complex than Th2 responses [66]. This information might partially elucidate the discrepancy observed in obtained results.

Note, although the conversion of some lineages of the Th cell into another is thought to be a one-way street (Treg to Th1), it is unclear whether unidirectionally convert perception is true [151]. The remarkable phenotypic plasticity discovered in Th cell may indicate that there is only a single T cell population that, depending on the environmental stimuli and the cytokine milieu resulting from the initial function of innate immune cells and in the continuation of acquired immune cells, undergo a global reprogramming that drives conversion to different functional properties [151]. According to this scenario, it might be better to focus the treatments on orchestrating the cytokines landscape, which could affect the selective expression of transcription factors that are presumably sensitive to environmental signals, referred to as “transdifferentiation,” instead of targeting a specific lineage of Th cell (such as Th17). Meanwhile, functional reprogramming has been suggested to establish homeostatic conditions [161]. Santos Rocha et al. have addressed that the modulation of pro- and anti-inflammatory cytokines is an important mechanism underlying the effects of several probiotics, which could be one of the main considerations influencing the function and potential therapeutic efficacy of MSCs [91]. Based on their milieu, the influence of MSCs on the T cell lineage seems diverse. Several studies have pointed to a potential for MSCs to suppress Th1 cells in vivo; others showed, on the contrary, that MSCs augmented Th1 responses [104, 162]. It was also reported in the case of humoral responses followed by MSC infusion. We found that a decrease in the percentage of Th1 and Th2 cells in the TCD4 + -cell population accompanied the alleviation of SLE-abnormalities in PIL mice by engrafted MSCs. Therefore, our data indicate that the engrafted MSCs directly affect both humoral and cellular responses, which implies that the therapeutic effect of MSCs regarding the reduction of autoantibodies could be, to some extent, due to their impact on Th cell subpopulations. Indeed, our results suggested that probiotic-trained MSCs suppressed TCD4^+^ cells from proliferating and changed committing to the Th cell fate. Obtained results suggested that R-MSCs treatment may have a more favorable outcome in altering the Th cell’s immunophenotype. However, D-MSCs treatment may reduce the proportion of Th cells more favorably. Researchers have already documented that bacteria-MSCs interactions can trigger and induce a phenotypic shift in MSCs [1, 163, 164]. Compatible with multiple reports, each bacteria has a different ability to change the immunomodulation capacity and biological behavior of MSCs with a distinctively different phenotype [1]. While L. delbrueckii provoked MSCs to suppress the percentage of Th cell subpopulations more strongly, L. rhamnose was found to be more effective in provoking MSCs to suppress inflammation. Paradoxically, in mice treated with D-MSCs, despite a significant reduction in the percentage of Th1 and Th17, no significant change was observed in the serum level of IFN-ɣ and IL-17. As shown in similar conditions, the failure of an apparent difference with or without D-MSC in the production of IFN-γ and IL-17 suggests that the production of these cytokines by lymphoid cells other than Th1 and Th17 cells is an important issue to be addressed [66]. Increasing evidence points out that microbiome sensors and metabolic factors in the microenvironment can profoundly influence cell differentiation and response to immune stimuli and have immune-modifying potential [161]. A recent study by Montuori-Andrade et al. has shown the ability of L. delbrueckii to inhibit the inflammatory response, the numbers of inflammatory immunocytes, and autoantibody levels while increasing the IFN-γ/IL-4 cytokine ratio using a murine model of autoimmunity [165]. Furthermore, it should be noted that under the influence of the cytokine microenvironment, regulatory cell subsets can preserve their immunosuppressive function while losing their anti-inflammatory function [150]. How can we conciliate the apparent paradox of MSCs behaving as immune suppressor cells and conditional immune activators? A study by Romieu-Mourez and colleagues reported that cell culture parameters, such as cell density, serum factors, and TGF-β could readily manipulate the immune plasticity of MSCs, leading to unpredictable outcomes in clinical trials with MSC-based therapies [166, 167]. On the other hand, probiotics have exhibited the ability to restore immune tolerance by releasing inhibitory cytokines, such as TGF‐β, inconsistent with the literature [168]. We found that our MSCs supported the expansion of Foxp3^+^ cell populations. However, we failed to demonstrate the subpopulations in TCD4^+^ IL-17^+^, TCD4^+^ IFN-ɣ^+^, and TCD4^+^ FOXP3^+^ that should accompany by more information. It may be possible to propose this hypothesis that the source of lupus-induced Th cells, inflammatory or suppressive phenotypes, is influenced by the overlap between Th cell subpopulations due to the flexibility of Th cells based on their environment and de novo transcription factor expression. In the following, many studies have demonstrated a relationship between MSCs’ immune plasticity and their microenvironment [7, 169].

In summary, the initiation and development of autoimmunity and lupus nephritis depend on complex multigenic interactions [170–172]. This study aimed to investigate the effect of BM-derived MSCs exposed to the tolerogenic probiotics in a murine model of SLE— Pristane-induced lupus mice in BALB/c background. Under the training in probiotics, MSCs showed different capacities/mechanisms in modulating immune hemostasis. In the present study, we distinguished that probiotic L. rhamnosus interactions with naïve MSCs could induce a new generation of MSCs with higher capacity in modulating inflammatory agents. Moreover, results revealed that naïve MSCs pretreated with L. delbrueckii had higher suppressive properties on cell proliferation than naïve MSCs. However, compared to the individual probiotic, naïve MSCs pretreated with a mixed of both probiotics appeared differently.

The discrepancy in obtained results is not surprising because MSCs (R-MSCs, D-MSCs, DR-MSCs) are known to will be differ functionally depending on their surrounding milieu and activating stimuli [166]. It is reported that MSCs are at rest state and require a “licensing” step to get active. Some research groups that have hypothesized the opposite regulation of IFN-ɣ and TGF-β induced immune responses have speculated that TGF-β pretreatment may further skew the immune phenotype of MSCs toward increased immunosuppression, thereby affecting the outcome of their infusion in vivo [166]. On the other hand, other studies have shown an increase in TGF-β regulating cytokine production after ingesting some probiotic species [91, 173–175]. L. rhamnosus and L. delbrueckii might have evolved different mechanisms to regulate host immune systems comprising the downregulation of inflammatory cytokines gene expression, degradation of mRNA and/or proteins, cell apoptosis, inhibiting immune cell proliferation, regulating the immune plasticity of MSC, disrupting signaling pathways through cytokine, and other mechanisms that have remained undefined [46, 176, 177]. Interest in developing new research initiatives exploring the physiological functions of probiotics that are highly strain-specific has increased over the past decade [178]. A hypothesis, R-MSC, may induce T cell tolerance [90].

It is now known that the organisms may behave differently when administered as a single strain versus as a combination of probiotic strains, potentiating or inhibiting the activity of each other [178]. We speculate that exposure to a mix of them can act as a new microenvironment, possibly containing and/or different ratios of certain elements, which licenses MSCs to act relatively differently. Future work is needed to identify the mechanism(s), such as epigenetic modifications and signaling pathways induced by bacteria. Under training in various Lactobacillus strains, MSCs can acquire different capacities in modulating immune hemostasis. It seems necessary to perform specific screenings to select appropriate probiotic strains for each condition that may become a novel treatment approach. It is not far from the mind that a particular generation of MSCs with high immunoregulatory capability might result in removing inflammatory conditions in locally and systemically therapeutic strategies. While the use of many immunosuppressive factors is frequently associated with side effects, the use of probiotic-educated MSCs is considered safe and well-tolerated. We hypothesized here that probiotic-educated MSCs could offer some advantages, such as promoting the local release of factors that may synergistically promote repair processes and immunomodulation, reducing inflammation, tissue damage, and nephritis.

## Conclusion

Co-culturing probiotics with MSCs showed distinct effects from the individual MSCs on lupus-liked symptoms. Our findings suggest that probiotics may exert potent effects on the immune regulatory capability of MSCs and, consequently in MSC-based treatment strategies. However, our understanding of the mechanism remained restricted. Taking into account the notable significance and extensive investigations regarding probiotics and MSCs and their function in the context of lupus, changes in MSCs transcriptome or protein function are important and need more extensive studies. Future studies are needed to uncover how MSCs play a therapeutic role in vivo after pretreatment and the corresponding molecular mechanisms are worth exploring. Moreover, particular randomized controlled trials are warranted to confirm the efficacy and safety of Lactobacillus-trained MSCs in patients with SLE. Our perspective is that the investigation into whether Lactobacillus will be a friend or foe in the context of SLE is contingent on the strain itself and the microenvironment. Moreover, different strains of Lactobacillus pose different effects even may opposite. Consequently, the interplay between different strains to produce an effective would also be another focusing point.

All and all, current evidence indicates that such immunomodulation without triggering inflammatory responses induced by L. rhamnosus in MSCs (termed as R-MSCs) makes them a good candidate for the preconditioning agent in opposing the progression of systemic and organ-specific autoinflammatory diseases. On the other hand, the default state of the tumor microenvironment is chronic inflammation, where the activity of tumor cells modulates molecular signaling and regulatory mechanisms predominate. Importantly, IFN-γ and IL-17 as functional mediators in antitumor immunity have been observed [150]. According to the tumor microenvironment, DR-MSCs are proposed as rehabilitation research in cancer disease that may activate inflammatory responses. Therefore, considering the clinical studies conducted worldwide using MSCs or probiotics alone, it can be concluded that the directed accelerated immunomodulatory capability of coincubated MSCs with probiotics in cellular therapies requires further consideration and examination to verify their effectiveness. Since the effect of probiotic use is expressly dependent upon the strain, it could also be suggested that more assessment is needed to select strains that provide the most helpful immunotherapy. The microbial immunomodulating approach by probiotics highlights that the new generation of MSC may represent an attractive cellular therapy for the future. The complex signaling pathways and molecules involved in changing MSCs’ plasticity in favor of improved curative effects may become a research hotspot in many diseases. Furthermore, flow cytometric analysis and cell sorting of digested kidney and liver tissue samples of PIL mice treated with naïve MSCs and pretreated probiotic-pretreated MSCs are highly desired [90].

## Data Availability

The data will be made available on request.

## Abbreviations

ANOVA: Analysis of variance
AC: Apoptotic cells
BM: Bone marrow
C3: Complement component 3
CFU-F: Colony-forming unit fibroblast
D-MSC: Delbrueckii-trained MSC
DMEM: Dulbecco’s modified Eagle’s medium
dsDNA: Double-Stranded DNA
DR-MSC: Delbrueckii/Rhamnose-trained MSC
EAE: Experimental allergic encephalomyelitis
ELISA: Enzyme-linked immunosorbent assay
FAO: Food and Agriculture Organization of the United Nations
FOXP3: Forkhead box P3
IFN‐γ: Interferon‐gamma
Ig: Immunoglobulin
IL: Interleukin
MRS: De Man, Rogosa, and Sharpe
MSC: Mesenchymal Stem/Stromal Cells
Naïve MSC: N-MSC
PBS: Phosphate buffered saline
PIL: Pristane-induced lupus
R-MSC: Rhamnose-trained MSC
RORγ: Retinoid-related orphan receptor γ
SEM: Standard deviation
SLE: Systemic lupus erythematosus
Th: T helper cell
TNF-α: Tumor Necrosis Factor alpha
Treg: Regulatory T cells
WHO: World Health Organization
TGF-β: Transforming growth factor-β
